# Supplementary material for: A Postsynaptic Density Immediate Early Gene-Based Connectome Analysis of Acute NMDAR Blockade and Reversal Effect of Antipsychotic Administration
Source: Int J Mol Sci. 2023 Feb 22;24(5):4372. doi: 10.3390/ijms24054372 (PMC10002165; doi:10.3390/ijms24054372)
Supplement: Supplementary file 1 [file ijms-24-04372-s001.zip › ijms-2209535-supplementary.pdf]

Supplementary Material

Supplementary Table S1. Results of Pearson's r correlations in VEH/VEH rats. Correlation coefficients and p-values for all possible pairs. Significant values were given in bold.

ROI	ROI	Pearson's *r*	p-value
Ig	cg2	0.884	**0.047**
Ig	cg1	0.921	**0.026**
cg2	cg1	0.983	**0.003**
Ig	M2	0.842	0.074
cg2	M2	0.915	**0.029**
cg1	M2	0.964	**0.008**
Ig	M1	0.756	0.139
cg2	M1	0.831	0.081
cg1	M1	0.898	**0.039**
M2	M1	0.974	**0.005**
Ig	S1FL	0.590	0.295
cg2	S1FL	0.750	0.144
cg1	S1FL	0.811	0.096
M2	S1FL	0.928	**0.023**
M1	S1FL	0.968	**0.007**
Ig	S1j	0.811	0.096
cg2	S1j	0.948	**0.014**
cg1	S1j	0.967	**0.007**
M2	S1j	0.980	**0.003**
M1	S1j	0.959	**0.010**
S1FL	S1j	0.916	**0.029**
Ig	S1JO	0.167	0.788
cg2	S1JO	0.396	0.509
cg1	S1JO	0.344	0.571
M2	S1JO	0.313	0.608
M1	S1JO	0.148	0.812
S1FL	S1JO	0.252	0.683
S1j	S1JO	0.290	0.636
Ig	S1DZ	0.797	0.106
cg2	S1DZ	0.973	**0.005**
cg1	S1DZ	0.943	**0.016**
M2	S1DZ	0.887	**0.045**
M1	S1DZ	0.843	0.073
S1FL	S1DZ	0.778	0.121
S1j	S1DZ	0.956	**0.011**
S1JO	S1DZ	0.279	0.649
Ig	S1ULp	0.614	0.271
cg2	S1ULp	0.873	0.053
cg1	S1ULp	0.788	0.113
M2	S1ULp	0.687	0.200
M1	S1ULp	0.639	0.246

ROI	ROI	Pearson's *r*	p-value
cg2	GI	0.869	0.056
cg1	GI	0.798	0.106
M2	GI	0.725	0.166
M1	GI	0.700	0.188
S1FL	GI	0.663	0.223
S1j	GI	0.847	0.070
S1JO	GI	0.213	0.731
S1DZ	GI	0.953	**0.012**
S1ULp	GI	0.993	**0.001**
Ig	DI	0.732	0.160
cg2	DI	0.962	**0.009**
cg1	DI	0.905	**0.035**
M2	DI	0.827	0.084
M1	DI	0.751	0.143
S1FL	DI	0.710	0.179
S1j	DI	0.907	**0.034**
S1JO	DI	0.434	0.466
S1DZ	DI	0.979	**0.004**
S1ULp	DI	0.961	**0.009**
GI	DI	0.954	**0.012**
Ig	AID	0.518	0.371
cg2	AID	0.823	0.087
cg1	AID	0.805	0.101
M2	AID	0.848	0.069
M1	AID	0.857	0.063
S1FL	AID	0.903	**0.036**
S1j	AID	0.915	**0.029**
S1JO	AID	0.361	0.550
S1DZ	AID	0.893	**0.041**
S1ULp	AID	0.838	0.076
GI	AID	0.883	0.047
DI	AID	0.884	**0.046**
Ig	AIV	0.723	0.168
cg2	AIV	0.843	0.073
cg1	AIV	0.885	**0.046**
M2	AIV	0.933	**0.021**
M1	AIV	0.864	0.059
S1FL	AIV	0.875	0.052
S1j	AIV	0.886	**0.045**
S1JO	AIV	0.611	0.274
S1DZ	AIV	0.770	0.128

S1FL	S1ULp	0.591	0.294
S1j	S1ULp	0.812	0.095
S1JO	S1ULp	0.261	0.671
S1DZ	S1ULp	0.942	**0.017**
Ig	GI	0.604	0.281
cg1	CI	0.773	0.125
M2	CI	0.893	**0.041**
M1	CI	0.877	0.051
S1FL	CI	0.929	**0.023**
S1j	CI	0.828	0.084
S1JO	CI	0.505	0.385
S1DZ	CI	0.649	0.236
S1ULp	CI	0.416	0.486
GI	CI	0.469	0.426
DI	CI	0.617	0.267
AID	CI	0.780	0.120
AIV	CI	0.959	**0.010**
Ig	Pir	0.522	0.367
cg2	Pir	0.399	0.506
cg1	Pir	0.551	0.336
M2	Pir	0.715	0.174
M1	Pir	0.746	0.148
S1FL	Pir	0.745	0.149
S1j	Pir	0.574	0.311
S1JO	Pir	0.146	0.814
S1DZ	Pir	0.315	0.606
S1ULp	Pir	-0.011	0.986
GI	Pir	0.058	0.926
DI	Pir	0.208	0.737
AID	Pir	0.398	0.507
AIV	Pir	0.729	0.162
CI	Pir	0.841	0.074
Ig	Den	0.531	0.357
cg2	Den	0.724	0.167
cg1	Den	0.780	0.120
M2	Den	0.903	**0.036**
M1	Den	0.926	**0.024**
S1FL	Den	0.987	**0.002**
S1j	Den	0.884	**0.047**
S1JO	Den	0.384	0.523
S1DZ	Den	0.737	0.155
S1ULp	Den	0.554	0.333
GI	Den	0.620	0.264
DI	Den	0.695	0.193
AID	Den	0.899	**0.038**
AIV	Den	0.907	**0.033**
CI	Den	0.962	**0.009**

S1ULp	AIV	0.567	0.318
GI	AIV	0.590	0.295
DI	AIV	0.766	0.131
AID	AIV	0.798	0.105
Ig	CI	0.579	0.307
M1	LSS	0.835	0.078
S1FL	LSS	0.940	**0.017**
S1j	LSS	0.811	0.095
S1JO	LSS	0.499	0.392
S1DZ	LSS	0.674	0.213
S1ULp	LSS	0.527	0.362
GI	LSS	0.589	0.297
DI	LSS	0.665	0.221
AID	LSS	0.895	**0.040**
AIV	LSS	0.880	**0.049**
CI	LSS	0.941	**0.017**
Pir	LSS	0.661	0.225
Den	LSS	0.980	**0.003**
Ig	CPDM	0.475	0.418
cg2	CPDM	0.606	0.279
cg1	CPDM	0.689	0.198
M2	CPDM	0.839	0.075
M1	CPDM	0.847	0.070
S1FL	CPDM	0.923	**0.025**
S1j	CPDM	0.770	0.128
S1JO	CPDM	0.467	0.428
S1DZ	CPDM	0.570	0.316
S1ULp	CPDM	0.336	0.580
GI	CPDM	0.401	0.504
DI	CPDM	0.533	0.355
AID	CPDM	0.754	0.141
AIV	CPDM	0.913	**0.031**
CI	CPDM	0.991	**0.001**
Pir	CPDM	0.855	0.065
Den	CPDM	0.961	**0.009**
LSS	CPDM	0.950	**0.013**
Ig	CPDL	0.612	0.273
cg2	CPDL	0.759	0.137
cg1	CPDL	0.823	0.087
M2	CPDL	0.933	**0.021**
M1	CPDL	0.931	**0.021**
S1FL	CPDL	0.973	**0.005**
S1j	CPDL	0.893	**0.041**
S1JO	CPDL	0.435	0.464
S1DZ	CPDL	0.739	0.154
S1ULp	CPDL	0.528	0.360
GI	CPDL	0.585	0.300

Pir	Den	0.744	0.149
Ig	LSS	0.394	0.512
cg2	LSS	0.655	0.230
cg1	LSS	0.691	0.196
M2	LSS	0.821	0.089
Den	CPDL	0.989	**0.001**
LSS	CPDL	0.958	**0.010**
CPDM	CPDL	0.974	**0.005**
Ig	CPVL	0.695	0.192
cg2	CPVL	0.802	0.103
cg1	CPVL	0.870	0.055
M2	CPVL	0.965	**0.008**
M1	CPVL	0.959	**0.010**
S1FL	CPVL	0.971	**0.006**
S1j	CPVL	0.922	**0.026**
S1JO	CPVL	0.386	0.521
S1DZ	CPVL	0.774	0.124
S1ULp	CPVL	0.551	0.336
GI	CPVL	0.605	0.280
DI	CPVL	0.720	0.170
AID	CPVL	0.845	0.072
AIV	CPVL	0.958	**0.010**
CI	CPVL	0.976	**0.004**
Pir	CPVL	0.811	0.096
Den	CPVL	0.973	**0.005**
LSS	CPVL	0.921	**0.027**
CPDM	CPVL	0.952	**0.012**
CPDL	CPVL	0.993	**0.001**
Ig	CPVM	0.605	0.280
cg2	CPVM	0.675	0.211
cg1	CPVM	0.771	0.127
M2	CPVM	0.909	**0.033**
M1	CPVM	0.930	**0.022**
S1FL	CPVM	0.958	**0.010**
S1j	CPVM	0.841	0.074
S1JO	CPVM	0.314	0.607
S1DZ	CPVM	0.646	0.239
S1ULp	CPVM	0.392	0.514
GI	CPVM	0.461	0.435
DI	CPVM	0.574	0.312
AID	CPVM	0.765	0.132
AIV	CPVM	0.909	**0.033**
CI	CPVM	0.978	**0.004**
Pir	CPVM	0.894	**0.041**
Den	CPVM	0.962	**0.009**
LSS	CPVM	0.909	**0.032**
CPDM	CPVM	0.976	**0.005**

DI	CPDL	0.698	0.190
AID	CPDL	0.859	0.062
AIV	CPDL	0.953	**0.012**
CI	CPDL	0.988	**0.002**
Pir	CPDL	0.796	0.107
M2	AcCo	0.761	0.135
M1	AcCo	0.782	0.118
S1FL	AcCo	0.892	**0.042**
S1j	AcCo	0.695	0.193
S1JO	AcCo	0.479	0.415
S1DZ	AcCo	0.487	0.405
S1ULp	AcCo	0.273	0.657
GI	AcCo	0.342	0.573
DI	AcCo	0.462	0.434
AID	AcCo	0.731	0.161
AIV	AcCo	0.859	0.062
CI	AcCo	0.963	**0.008**
Pir	AcCo	0.817	0.092
Den	AcCo	0.942	**0.017**
LSS	AcCo	0.955	**0.011**
CPDM	AcCo	0.989	**0.001**
CPDL	AcCo	0.942	**0.017**
CPVL	AcCo	0.904	**0.035**
CPVM	AcCo	0.941	**0.017**
Ig	AcSh	0.452	0.444
cg2	AcSh	0.627	0.257
cg1	AcSh	0.695	0.193
M2	AcSh	0.838	0.076
M1	AcSh	0.842	0.073
S1FL	AcSh	0.930	**0.022**
S1j	AcSh	0.785	0.116
S1JO	AcSh	0.507	0.383
S1DZ	AcSh	0.604	0.281
S1ULp	AcSh	0.396	0.509
GI	AcSh	0.458	0.438
DI	AcSh	0.581	0.304
AID	AcSh	0.801	0.103
AIV	AcSh	0.917	**0.028**
CI	AcSh	0.987	**0.002**
Pir	AcSh	0.798	0.105
Den	AcSh	0.973	**0.005**
LSS	AcSh	0.975	**0.005**
CPDM	AcSh	0.995	**<0.001**
CPDL	AcSh	0.978	**0.004**
CPVL	AcSh	0.949	**0.014**
CPVM	AcSh	0.961	**0.009**
AcCo	AcSh	0.990	**0.001**

CPDL	CPVM	0.980	**0.003**
CPVL	CPVM	0.981	**0.003**
Ig	AcCo	0.343	0.572
cg2	AcCo	0.509	0.381
cg1	AcCo	0.589	0.296
S1FL	LSD	0.218	0.724
S1j	LSD	0.420	0.482
S1JO	LSD	0.571	0.314
S1DZ	LSD	0.389	0.518
S1ULp	LSD	0.213	0.730
GI	LSD	0.153	0.805
DI	LSD	0.421	0.480
AID	LSD	0.130	0.835
AIV	LSD	0.625	0.260
CI	LSD	0.450	0.448
Pir	LSD	0.420	0.481
Den	LSD	0.250	0.685
LSS	LSD	0.191	0.759
CPDM	LSD	0.348	0.565
CPDL	LSD	0.384	0.523
CPVL	LSD	0.439	0.459
CPVM	LSD	0.369	0.542
AcCo	LSD	0.246	0.690
AcSh	LSD	0.319	0.601
Ig	LSI	0.842	0.074
cg2	LSI	0.786	0.115
cg1	LSI	0.778	0.121
M2	LSI	0.650	0.235
M1	LSI	0.468	0.426
S1FL	LSI	0.353	0.560
S1j	LSI	0.607	0.278
S1JO	LSI	0.599	0.285
S1DZ	LSI	0.628	0.257
S1ULp	LSI	0.499	0.392
GI	LSI	0.441	0.458
DI	LSI	0.668	0.217
AID	LSI	0.363	0.548
AIV	LSI	0.715	0.175
CI	LSI	0.507	0.383
Pir	LSI	0.340	0.575
Den	LSI	0.372	0.538
LSS	LSI	0.316	0.605
CPDM	LSI	0.391	0.515
CPDL	LSI	0.483	0.410
CPVL	LSI	0.538	0.349
CPVM	LSI	0.423	0.478
AcCo	LSI	0.282	0.646
Ig	LSD	0.750	0.144
cg2	LSD	0.591	0.293
cg1	LSD	0.618	0.267
M2	LSD	0.516	0.374
M1	LSD	0.332	0.585
M2	LSV	0.699	0.189
M1	LSV	0.548	0.339
S1FL	LSV	0.520	0.369
S1j	LSV	0.600	0.285
S1JO	LSV	0.740	0.153
S1DZ	LSV	0.499	0.392
S1ULp	LSV	0.288	0.638
GI	LSV	0.262	0.670
DI	LSV	0.536	0.352
AID	LSV	0.410	0.493
AIV	LSV	0.859	0.062
CI	LSV	0.754	0.141
Pir	LSV	0.630	0.255
Den	LSV	0.581	0.304
LSS	LSV	0.559	0.327
CPDM	LSV	0.684	0.202
CPDL	LSV	0.687	0.200
CPVL	LSV	0.707	0.182
CPVM	LSV	0.659	0.227
AcCo	LSV	0.615	0.270
AcSh	LSV	0.668	0.218
LSD	LSV	0.908	**0.033**
LSI	LSV	0.889	**0.044**
Ig	Shi	0.830	0.082
cg2	Shi	0.890	**0.043**
cg1	Shi	0.882	**0.048**
M2	Shi	0.803	0.102
M1	Shi	0.648	0.237
S1FL	Shi	0.586	0.299
S1j	Shi	0.776	0.123
S1JO	Shi	0.687	0.200
S1DZ	Shi	0.768	0.129
S1ULp	Shi	0.634	0.251
GI	Shi	0.599	0.286
DI	Shi	0.810	0.097
AID	Shi	0.611	0.274
AIV	Shi	0.877	0.051
CI	Shi	0.708	0.181
Pir	Shi	0.445	0.453
Den	Shi	0.616	0.269
LSS	Shi	0.581	0.304
CPDM	Shi	0.610	0.275

AcSh	LSI	0.386	0.522
LSD	LSI	0.952	**0.012**
Ig	LSV	0.698	0.190
cg2	LSV	0.674	0.212
cg1	LSV	0.715	0.174
AcSh	Shi	0.619	0.265
LSD	Shi	0.856	0.064
LSI	Shi	0.953	**0.012**
LSV	Shi	0.916	**0.029**
Ig	MS	0.758	0.137
cg2	MS	0.824	0.087
cg1	MS	0.844	0.073
M2	MS	0.822	0.087
M1	MS	0.686	0.201
S1FL	MS	0.664	0.222
S1j	MS	0.765	0.132
S1JO	MS	0.740	0.153
S1DZ	MS	0.693	0.195
S1ULp	MS	0.513	0.377
GI	MS	0.496	0.396
DI	MS	0.728	0.163
AID	MS	0.621	0.264
AIV	MS	0.941	**0.017**
CI	MS	0.826	0.085
Pir	MS	0.598	0.287
Den	MS	0.713	0.176
LSS	MS	0.693	0.195
CPDM	MS	0.752	0.143
CPDL	MS	0.795	0.108
CPVL	MS	0.812	0.095
CPVM	MS	0.736	0.156
AcCo	MS	0.681	0.206
AcSh	MS	0.754	0.141
LSD	MS	0.832	0.081
LSI	MS	0.890	**0.043**
LSV	MS	0.966	**0.007**
Shi	MS	0.971	**0.006**
Ig	VDB	0.237	0.701
cg2	VDB	0.381	0.527
cg1	VDB	0.456	0.441
M2	VDB	0.615	0.270
M1	VDB	0.592	0.293
S1FL	VDB	0.722	0.168
S1j	VDB	0.520	0.370
S1JO	VDB	0.655	0.230
S1DZ	VDB	0.306	0.617
S1ULp	VDB	0.095	0.880

CPDL	Shi	0.699	0.189
CPVL	Shi	0.732	0.160
CPVM	Shi	0.619	0.265
AcCo	Shi	0.520	0.370
AcSh	Shi	0.619	0.265
Pir	VDB	0.770	0.128
Den	VDB	0.812	0.095
LSS	VDB	0.858	0.063
CPDM	VDB	0.928	**0.023**
CPDL	VDB	0.836	0.077
CPVL	VDB	0.787	0.114
CPVM	VDB	0.832	0.081
AcCo	VDB	0.950	**0.013**
AcSh	VDB	0.925	**0.025**
LSD	VDB	0.362	0.549
LSI	VDB	0.336	0.581
LSV	VDB	0.709	0.180
Shi	VDB	0.533	0.355
MS	VDB	0.714	0.176
Ig	Icj	0.785	0.116
cg2	Icj	0.930	**0.022**
cg1	Icj	0.885	**0.046**
M2	Icj	0.792	0.111
M1	Icj	0.752	0.143
S1FL	Icj	0.653	0.232
S1j	Icj	0.883	0.047
S1JO	Icj	0.134	0.830
S1DZ	Icj	0.976	**0.004**
S1ULp	Icj	0.960	**0.009**
GI	Icj	0.962	**0.009**
DI	Icj	0.947	**0.014**
AID	Icj	0.806	0.099
AIV	Icj	0.620	0.264
CI	Icj	0.475	0.419
Pir	Icj	0.162	0.795
Den	Icj	0.588	0.297
LSS	Icj	0.508	0.382
CPDM	Icj	0.387	0.520
CPDL	Icj	0.584	0.301
CPVL	Icj	0.633	0.251
CPVM	Icj	0.491	0.401
AcCo	Icj	0.295	0.630
AcSh	Icj	0.422	0.479
LSD	Icj	0.325	0.594
LSI	Icj	0.579	0.306
LSV	Icj	0.360	0.552
Shi	Icj	0.680	0.206

GI	VDB	0.141	0.821
DI	VDB	0.323	0.596
AID	VDB	0.553	0.333
AIV	VDB	0.813	0.094
CI	VDB	0.902	**0.036**
M2	VP	0.609	0.276
M1	VP	0.719	0.171
S1FL	VP	0.824	0.086
S1j	VP	0.534	0.353
S1JO	VP	0.086	0.890
S1DZ	VP	0.287	0.640
S1ULp	VP	0.058	0.927
GI	VP	0.162	0.795
DI	VP	0.194	0.754
AID	VP	0.575	0.311
AIV	VP	0.617	0.268
CI	VP	0.813	0.094
Pir	VP	0.840	0.075
Den	VP	0.835	0.079
LSS	VP	0.816	0.092
CPDM	VP	0.878	**<0.050**
CPDL	VP	0.806	0.100
CPVL	VP	0.772	0.126
CPVM	VP	0.869	0.056
AcCo	VP	0.905	**0.035**
AcSh	VP	0.858	0.063
LSD	VP	-0.057	0.927
LSI	VP	-0.061	0.922
LSV	VP	0.301	0.622
Shi	VP	0.169	0.785
MS	VP	0.353	0.560
VDB	VP	0.796	0.107
Icj	VP	0.124	0.843
Ig	Tu	0.766	0.131
cg2	Tu	0.498	0.394
cg1	Tu	0.552	0.335
M2	Tu	0.456	0.440
M1	Tu	0.300	0.624
S1FL	Tu	0.144	0.817
S1j	Tu	0.341	0.574
S1JO	Tu	0.347	0.567
S1DZ	Tu	0.294	0.632
S1ULp	Tu	0.088	0.888
GI	Tu	0.036	0.954
DI	Tu	0.283	0.645
AID	Tu	-0.014	0.982
AIV	Tu	0.506	0.385
MS	Icj	0.561	0.325
VDB	Icj	0.092	0.883
Ig	VP	0.172	0.781
cg2	VP	0.258	0.675
cg1	VP	0.378	0.531
CPDL	Tu	0.287	0.640
CPVL	Tu	0.362	0.550
CPVM	Tu	0.316	0.604
AcCo	Tu	0.138	0.825
AcSh	Tu	0.203	0.744
LSD	Tu	0.965	**0.008**
LSI	Tu	0.878	**<0.050**
LSV	Tu	0.812	0.095
Shi	Tu	0.736	0.156
MS	Tu	0.706	0.182
VDB	Tu	0.234	0.705
Icj	Tu	0.258	0.676
VP	Tu	-0.077	0.902

CI	Tu	0.351	0.562
Pir	Tu	0.457	0.439
Den	Tu	0.145	0.816
LSS	Tu	0.046	0.941
CPDM	Tu	0.254	0.681

Supplementary Table S2. Results of Pearson's r correlations in VEH/ASE rats. Correlation coefficients and p-values for all possible pairs. Significant values were given in bold.

ROI	ROI	Pearson's r	p-value
Ig	cg2	0.482	0.411
Ig	cg1	0.319	0.601
cg2	cg1	0.276	0.654
Ig	M2	-0.394	0.512
cg2	M2	0.057	0.927
cg1	M2	0.514	0.376
Ig	M1	-0.591	0.294
cg2	M1	0.371	0.539
cg1	M1	-0.195	0.753
M2	M1	0.534	0.354
Ig	S1FL	-0.163	0.794
cg2	S1FL	0.270	0.661
cg1	S1FL	0.403	0.501
M2	S1FL	-0.025	0.968
M1	S1FL	0.122	0.845
Ig	S1j	-0.513	0.377
cg2	S1j	-0.753	0.142
cg1	S1j	0.224	0.717
M2	S1j	0.126	0.840
M1	S1j	-0.296	0.628
S1FL	S1j	0.379	0.529
Ig	S1JO	-0.383	0.524
cg2	S1JO	-0.925	**0.025**
cg1	S1JO	-0.386	0.521
M2	S1JO	-0.390	0.517
M1	S1JO	-0.481	0.412
S1FL	S1JO	-0.069	0.912
S1j	S1JO	0.740	0.153
Ig	S1DZ	0.735	0.157
cg2	S1DZ	-0.030	0.962
cg1	S1DZ	0.420	0.481
M2	S1DZ	-0.510	0.380
M1	S1DZ	-0.917	**0.028**
S1FL	S1DZ	0.173	0.781
S1j	S1DZ	0.182	0.769
S1JO	S1DZ	0.199	0.748
Ig	S1ULp	0.619	0.266
cg2	S1ULp	0.309	0.613
cg1	S1ULp	0.425	0.476
M2	S1ULp	-0.532	0.357
M1	S1ULp	-0.592	0.293
S1FL	S1ULp	0.603	0.282
S1j	S1ULp	0.089	0.887
S1JO	S1ULp	-0.031	0.961
S1DZ	S1ULp	0.846	0.071
Ig	GI	0.518	0.371
cg2	GI	-0.256	0.678
cg1	GI	0.182	0.770
M2	GI	-0.672	0.214
S1JO	CI	0.762	0.134
S1DZ	CI	0.403	0.501

ROI	ROI	Pearson's r	p-value
M1	GI	-0.923	**0.025**
S1FL	GI	0.227	0.714
S1j	GI	0.363	0.548
S1JO	GI	0.484	0.408
S1DZ	GI	0.938	**0.018**
S1ULp	GI	0.804	0.101
Ig	DI	0.301	0.623
cg2	DI	-0.151	0.809
cg1	DI	-0.162	0.795
M2	DI	-0.872	0.054
M1	DI	-0.644	0.241
S1FL	DI	0.408	0.495
S1j	DI	0.267	0.664
S1JO	DI	0.498	0.393
S1DZ	DI	0.695	0.193
S1ULp	DI	0.773	0.125
GI	DI	0.865	0.058
Ig	AID	-0.185	0.766
cg2	AID	0.390	0.517
cg1	AID	-0.446	0.452
M2	AID	-0.477	0.417
M1	AID	0.396	0.510
S1FL	AID	0.554	0.332
S1j	AID	-0.213	0.731
S1JO	AID	-0.092	0.882
S1DZ	AID	-0.216	0.727
S1ULp	AID	0.260	0.673
GI	AID	-0.057	0.927
DI	AID	0.432	0.467
Ig	AIV	0.713	0.177
cg2	AIV	0.580	0.305
cg1	AIV	-0.342	0.573
M2	AIV	-0.619	0.266
M1	AIV	-0.143	0.819
S1FL	AIV	-0.285	0.642
S1j	AIV	-0.845	0.071
S1JO	AIV	-0.400	0.504
S1DZ	AIV	0.235	0.704
S1ULp	AIV	0.288	0.639
GI	AIV	0.146	0.814
DI	AIV	0.268	0.663
AID	AIV	0.335	0.581
Ig	CI	0.152	0.807
cg2	CI	-0.604	0.281
cg1	CI	-0.595	0.290
M2	CI	-0.781	0.119
M1	CI	-0.665	0.221
S1FL	CI	-0.391	0.515
S1j	CI	0.173	0.780
AIV	LSS	0.619	0.266
CI	LSS	0.245	0.691

S1ULp	CI	0.119	0.848
GI	CI	0.609	0.275
DI	CI	0.634	0.250
AID	CI	-0.018	0.977
AIV	CI	0.269	0.661
Ig	Pir	0.143	0.819
cg2	Pir	0.375	0.533
cg1	Pir	-0.630	0.254
M2	Pir	-0.756	0.139
M1	Pir	0.127	0.839
S1FL	Pir	0.192	0.757
S1j	Pir	-0.462	0.434
S1JO	Pir	-0.056	0.928
S1DZ	Pir	-0.025	0.968
S1ULp	Pir	0.288	0.639
GI	Pir	0.109	0.861
DI	Pir	0.538	0.349
AID	Pir	0.878	**<0.050**
AIV	Pir	0.696	0.192
CI	Pir	0.311	0.611
Ig	Den	0.427	0.474
cg2	Den	-0.029	0.963
cg1	Den	-0.419	0.482
M2	Den	-0.993	**0.001**
M1	Den	-0.572	0.314
S1FL	Den	0.117	0.851
S1j	Den	-0.080	0.898
S1JO	Den	0.375	0.534
S1DZ	Den	0.581	0.304
S1ULp	Den	0.624	0.261
GI	Den	0.732	0.160
DI	Den	0.916	**0.029**
AID	Den	0.479	0.414
AIV	Den	0.589	0.296
CI	Den	0.741	0.152
Pir	Den	0.733	0.159
Ig	LSS	0.383	0.525
cg2	LSS	0.442	0.456
cg1	LSS	-0.224	0.718
M2	LSS	-0.792	0.110
M1	LSS	-0.172	0.782
S1FL	LSS	0.494	0.398
S1j	LSS	-0.270	0.661
S1JO	LSS	-0.068	0.913
S1DZ	LSS	0.388	0.519
S1ULp	LSS	0.719	0.171
GI	LSS	0.467	0.428
DI	LSS	0.775	0.124
AID	LSS	0.796	0.107
GI	CPVL	0.943	**0.016**
DI	CPVL	0.855	0.065
AID	CPVL	-0.067	0.915
AIV	CPVL	0.129	0.836
CI	CPVL	0.830	0.082
Pir	CPVL	0.156	0.802

Pir	LSS	0.870	0.055
Den	LSS	0.825	0.085
Ig	CPDM	-0.270	0.660
cg2	CPDM	-0.953	**0.012**
cg1	CPDM	-0.135	0.829
M2	CPDM	-0.218	0.724
M1	CPDM	-0.607	0.278
S1FL	CPDM	-0.136	0.827
S1j	CPDM	0.801	0.103
S1JO	CPDM	0.949	**0.014**
S1DZ	CPDM	0.323	0.596
S1ULp	CPDM	-0.012	0.985
GI	CPDM	0.532	0.356
DI	CPDM	0.384	0.523
AID	CPDM	-0.380	0.528
AIV	CPDM	-0.503	0.388
CI	CPDM	0.679	0.207
Pir	CPDM	-0.336	0.581
Den	CPDM	0.217	0.726
LSS	CPDM	-0.266	0.666
Ig	CPDL	-0.125	0.841
cg2	CPDL	-0.768	0.129
cg1	CPDL	-0.087	0.890
M2	CPDL	-0.474	0.420
M1	CPDL	-0.697	0.191
S1FL	CPDL	0.185	0.765
S1j	CPDL	0.790	0.112
S1JO	CPDL	0.914	**0.030**
S1DZ	CPDL	0.535	0.353
S1ULp	CPDL	0.357	0.555
GI	CPDL	0.764	0.132
DI	CPDL	0.713	0.176
AID	CPDL	-0.080	0.898
AIV	CPDL	-0.353	0.560
CI	CPDL	0.697	0.191
Pir	CPDL	-0.063	0.920
Den	CPDL	0.504	0.387
LSS	CPDL	0.127	0.838
CPDM	CPDL	0.910	**0.032**
Ig	CPVL	0.357	0.556
cg2	CPVL	-0.488	0.404
cg1	CPVL	-0.105	0.867
M2	CPVL	-0.752	0.143
M1	CPVL	-0.910	**0.032**
S1FL	CPVL	0.021	0.973
S1j	CPVL	0.403	0.501
S1JO	CPVL	0.702	0.187
S1DZ	CPVL	0.801	0.104
S1ULp	CPVL	0.585	0.300
CPVL	AcCo	0.473	0.421
CPVM	AcCo	0.591	0.294
Ig	AcSh	0.644	0.241
cg2	AcSh	0.729	0.162
cg1	AcSh	-0.288	0.638
M2	AcSh	-0.498	0.393

Den	CPVL	0.779	0.120
LSS	CPVL	0.382	0.526
CPDM	CPVL	0.705	0.184
CPDL	CPVL	0.863	0.059
Ig	CPVM	0.380	0.529
cg2	CPVM	-0.363	0.548
cg1	CPVM	-0.107	0.864
M2	CPVM	-0.819	0.090
M1	CPVM	-0.858	0.063
S1FL	CPVM	0.158	0.800
S1j	CPVM	0.352	0.562
S1JO	CPVM	0.631	0.253
S1DZ	CPVM	0.810	0.097
S1ULp	CPVM	0.689	0.198
GI	CPVM	0.956	**0.011**
DI	CPVM	0.934	**0.020**
AID	CPVM	0.097	0.876
AIV	CPVM	0.199	0.748
CI	CPVM	0.779	0.120
Pir	CPVM	0.292	0.633
Den	CPVM	0.855	0.065
LSS	CPVM	0.539	0.349
CPDM	CPVM	0.599	0.285
CPDL	CPVM	0.827	0.084
CPVL	CPVM	0.983	**0.003**
Ig	AcCo	0.308	0.614
cg2	AcCo	0.218	0.725
cg1	AcCo	-0.561	0.326
M2	AcCo	-0.928	**0.023**
M1	AcCo	-0.212	0.732
S1FL	AcCo	0.173	0.781
S1j	AcCo	-0.317	0.604
S1JO	AcCo	0.138	0.824
S1DZ	AcCo	0.281	0.647
S1ULp	AcCo	0.485	0.407
GI	AcCo	0.428	0.472
DI	AcCo	0.764	0.132
AID	AcCo	0.744	0.149
AIV	AcCo	0.703	0.185
CI	AcCo	0.538	0.350
Pir	AcCo	0.941	**0.017**
Den	AcCo	0.919	**0.027**
LSS	AcCo	0.917	**0.028**
CPDM	AcCo	-0.099	0.874
CPDL	AcCo	0.204	0.742
cg1	LSI	-0.594	0.291
M2	LSI	-0.266	0.665
M1	LSI	0.622	0.263
S1FL	LSI	0.406	0.498
S1j	LSI	-0.138	0.825
S1JO	LSI	-0.010	0.988
S1DZ	LSI	-0.539	0.349
S1ULp	LSI	-0.103	0.869
GI	LSI	-0.338	0.578
DI	LSI	0.177	0.776

M1	AcSh	0.049	0.937
S1FL	AcSh	-0.174	0.779
S1j	AcSh	-0.907	**0.033**
S1JO	AcSh	-0.559	0.327
S1DZ	AcSh	0.104	0.868
S1ULp	AcSh	0.256	0.678
GI	AcSh	-0.008	0.990
DI	AcSh	0.161	0.795
AID	AcSh	0.434	0.466
AIV	AcSh	0.975	**0.005**
CI	AcSh	0.068	0.913
Pir	AcSh	0.718	0.172
Den	AcSh	0.473	0.421
LSS	AcSh	0.629	0.255
CPDM	AcSh	-0.676	0.210
CPDL	AcSh	-0.512	0.378
CPVL	AcSh	-0.061	0.922
CPVM	AcSh	0.032	0.959
AcCo	AcSh	0.660	0.226
Ig	LSD	-0.467	0.428
cg2	LSD	0.161	0.795
cg1	LSD	-0.732	0.160
M2	LSD	-0.334	0.582
M1	LSD	0.592	0.292
S1FL	LSD	0.229	0.711
S1j	LSD	-0.225	0.716
S1JO	LSD	0.029	0.963
S1DZ	LSD	-0.566	0.320
S1ULp	LSD	-0.194	0.754
GI	LSD	-0.356	0.557
DI	LSD	0.158	0.799
AID	LSD	0.888	**0.044**
AIV	LSD	0.231	0.709
CI	LSD	0.059	0.924
Pir	LSD	0.792	0.110
Den	LSD	0.288	0.638
LSS	LSD	0.497	0.394
CPDM	LSD	-0.282	0.646
CPDL	LSD	-0.151	0.809
CPVL	LSD	-0.232	0.708
CPVM	LSD	-0.122	0.845
AcCo	LSD	0.594	0.291
AcSh	LSD	0.319	0.601
Ig	LSI	-0.500	0.392
cg2	LSI	0.211	0.734
cg1	Shi	-0.234	0.705
M2	Shi	-0.628	0.257
M1	Shi	0.016	0.979
S1FL	Shi	0.730	0.161
S1j	Shi	0.074	0.906
S1JO	Shi	0.128	0.838
S1DZ	Shi	0.180	0.772
S1ULp	Shi	0.597	0.288
GI	Shi	0.356	0.557
DI	Shi	0.744	0.149

AID	LSI	0.927	**0.023**
AIV	LSI	0.132	0.832
CI	LSI	-0.064	0.919
Pir	LSI	0.751	0.144
Den	LSI	0.240	0.698
LSS	LSI	0.523	0.366
CPDM	LSI	-0.310	0.612
CPDL	LSI	-0.139	0.824
CPVL	LSI	-0.263	0.669
CPVM	LSI	-0.136	0.828
AcCo	LSI	0.547	0.340
AcSh	LSI	0.245	0.691
LSD	LSI	0.981	**0.003**
Ig	LSV	-0.125	0.841
cg2	LSV	0.156	0.802
cg1	LSV	-0.536	0.352
M2	LSV	-0.707	0.182
M1	LSV	0.109	0.861
S1FL	LSV	0.495	0.396
S1j	LSV	-0.057	0.928
S1JO	LSV	0.192	0.757
S1DZ	LSV	-0.003	0.996
S1ULp	LSV	0.370	0.539
GI	LSV	0.218	0.725
DI	LSV	0.677	0.210
AID	LSV	0.943	**0.016**
AIV	LSV	0.343	0.572
CI	LSV	0.293	0.632
Pir	LSV	0.902	**0.036**
Den	LSV	0.709	0.180
LSS	LSV	0.858	0.063
CPDM	LSV	-0.096	0.878
CPDL	LSV	0.221	0.721
CPVL	LSV	0.258	0.676
CPVM	LSV	0.402	0.502
AcCo	LSV	0.869	0.056
AcSh	LSV	0.371	0.538
LSD	LSV	0.820	0.089
LSI	LSV	0.837	0.077
Ig	Shi	-0.039	0.951
cg2	Shi	0.233	0.707
LSV	MS	0.793	0.110
Shi	MS	0.877	0.051
Ig	VDB	-0.173	0.781
cg2	VDB	-0.614	0.270
cg1	VDB	-0.840	0.075
M2	VDB	-0.708	0.181
M1	VDB	-0.322	0.597
S1FL	VDB	-0.362	0.549
S1j	VDB	0.149	0.812
S1JO	VDB	0.767	0.131
S1DZ	VDB	0.036	0.954
S1ULp	VDB	-0.136	0.828
GI	VDB	0.315	0.606
DI	VDB	0.503	0.387

AID	Shi	0.897	**0.039**
AIV	Shi	0.230	0.710
CI	Shi	0.143	0.818
Pir	Shi	0.777	0.122
Den	Shi	0.668	0.217
LSS	Shi	0.892	**0.042**
CPDM	Shi	-0.105	0.867
CPDL	Shi	0.278	0.651
CPVL	Shi	0.298	0.626
CPVM	Shi	0.459	0.437
AcCo	Shi	0.780	0.119
AcSh	Shi	0.273	0.656
LSD	Shi	0.648	0.237
LSI	Shi	0.721	0.169
LSV	Shi	0.945	**0.016**
Ig	MS	0.328	0.590
cg2	MS	0.646	0.239
cg1	MS	-0.037	0.953
M2	MS	-0.555	0.332
M1	MS	0.049	0.938
S1FL	MS	0.646	0.239
S1j	MS	-0.322	0.597
S1JO	MS	-0.313	0.608
S1DZ	MS	0.259	0.674
S1ULp	MS	0.696	0.191
GI	MS	0.276	0.653
DI	MS	0.590	0.295
AID	MS	0.832	0.081
AIV	MS	0.535	0.353
CI	MS	-0.079	0.899
Pir	MS	0.793	0.110
Den	MS	0.602	0.283
LSS	MS	0.947	**0.015**
CPDM	MS	-0.491	0.401
CPDL	MS	-0.092	0.883
CPVL	MS	0.118	0.851
CPVM	MS	0.297	0.628
AcCo	MS	0.765	0.132
AcSh	MS	0.613	0.271
LSD	MS	0.500	0.391
LSI	MS	0.569	0.317
CPVL	Icj	0.547	0.340
CPVM	Icj	0.605	0.280
AcCo	Icj	0.381	0.527
AcSh	Icj	-0.422	0.479
LSD	Icj	0.352	0.562
LSI	Icj	0.427	0.474
LSV	Icj	0.640	0.245
Shi	Icj	0.708	0.181
MS	Icj	0.307	0.615
VDB	Icj	0.448	0.449
Ig	VP	0.663	0.223
cg2	VP	0.837	0.077
cg1	VP	0.354	0.559
M2	VP	-0.325	0.594

AID	VDB	0.208	0.737
AIV	VDB	0.197	0.751
CI	VDB	0.918	**0.028**
Pir	VDB	0.449	0.448
Den	VDB	0.642	0.243
LSS	VDB	0.227	0.713
CPDM	VDB	0.591	0.294
CPDL	VDB	0.580	0.305
CPVL	VDB	0.600	0.285
CPVM	VDB	0.566	0.320
AcCo	VDB	0.565	0.321
AcSh	VDB	0.044	0.944
LSD	VDB	0.399	0.506
LSI	VDB	0.271	0.659
LSV	VDB	0.460	0.436
Shi	VDB	0.235	0.703
MS	VDB	-0.065	0.917
Ig	Icj	-0.420	0.481
cg2	Icj	-0.477	0.416
cg1	Icj	-0.184	0.768
M2	Icj	-0.434	0.465
M1	Icj	-0.222	0.720
S1FL	Icj	0.619	0.265
S1j	Icj	0.727	0.164
S1JO	Icj	0.716	0.173
S1DZ	Icj	0.206	0.739
S1ULp	Icj	0.372	0.538
GI	Icj	0.493	0.399
DI	Icj	0.716	0.174
AID	Icj	0.463	0.432
AIV	Icj	-0.381	0.527
CI	Icj	0.379	0.530
Pir	Icj	0.269	0.662
Den	Icj	0.473	0.421
LSS	Icj	0.407	0.497
CPDM	Icj	0.573	0.312
CPDL	Icj	0.793	0.110
GI	Tu	0.276	0.654
DI	Tu	0.607	0.278
AID	Tu	0.840	0.075
AIV	Tu	0.137	0.826
CI	Tu	-0.149	0.811
Pir	Tu	0.628	0.256
Den	Tu	0.469	0.425
LSS	Tu	0.827	0.084
CPDM	Tu	-0.271	0.660
CPDL	Tu	0.124	0.842
CPVL	Tu	0.124	0.843
CPVM	Tu	0.298	0.627
AcCo	Tu	0.600	0.285
AcSh	Tu	0.230	0.710
LSD	Tu	0.520	0.369
LSI	Tu	0.641	0.244
LSV	Tu	0.812	0.095
Shi	Tu	0.948	**0.014**

M1	VP	-0.084	0.893
S1FL	VP	0.517	0.372
S1j	VP	-0.470	0.424
S1JO	VP	-0.608	0.277
S1DZ	VP	0.448	0.449
S1ULp	VP	0.774	0.125
GI	VP	0.293	0.632
DI	VP	0.379	0.530
AID	VP	0.465	0.431
AIV	VP	0.590	0.295
CI	VP	-0.287	0.639
Pir	VP	0.483	0.409
Den	VP	0.391	0.515
LSS	VP	0.753	0.142
CPDM	VP	-0.639	0.246
CPDL	VP	-0.295	0.630
CPVL	VP	0.030	0.962
CPVM	VP	0.177	0.775
AcCo	VP	0.489	0.404
AcSh	VP	0.668	0.218
LSD	VP	0.061	0.922
LSI	VP	0.139	0.824
LSV	VP	0.380	0.528
Shi	VP	0.543	0.345
MS	VP	0.862	0.060
VDB	VP	-0.424	0.477
Icj	VP	-0.077	0.903
Ig	Tu	-0.001	0.998
cg2	Tu	0.411	0.491
cg1	Tu	0.048	0.939
M2	Tu	-0.404	0.499
M1	Tu	0.110	0.861
S1FL	Tu	0.882	**0.048**
S1j	Tu	0.066	0.916
S1JO	Tu	-0.094	0.881
S1DZ	Tu	0.181	0.771
S1ULp	Tu	0.661	0.225

MS	Tu	0.906	**0.034**
VDB	Tu	-0.084	0.893
Icj	Tu	0.604	0.280
VP	Tu	0.678	0.209

Supplementary Table S3. Results of Pearson's r correlations in KET/VEH rats. Correlation coefficients and p-values for all possible pairs. Significant values were given in bold.

ROI	ROI	Pearson's r	p-value
Ig	cg2	-0.010	0.987
Ig	cg1	-0.757	0.139
cg2	cg1	0.331	0.586
Ig	M2	-0.732	0.160
cg2	M2	0.261	0.671
cg1	M2	0.987	**0.002**
Ig	M1	-0.551	0.335
cg2	M1	0.520	0.369
cg1	M1	0.923	**0.025**
M2	M1	0.940	**0.017**
Ig	S1FL	-0.911	**0.031**
cg2	S1FL	-0.225	0.716
cg1	S1FL	0.802	0.103
M2	S1FL	0.822	0.088
M1	S1FL	0.600	0.284
Ig	S1j	-0.854	0.065
cg2	S1j	-0.383	0.525
cg1	S1j	0.699	0.189
M2	S1j	0.739	0.154
M1	S1j	0.494	0.397
S1FL	S1j	0.984	**0.002**
Ig	S1JO	0.020	0.974
cg2	S1JO	0.731	0.161
cg1	S1JO	0.320	0.599
M2	S1JO	0.367	0.543
M1	S1JO	0.648	0.237
S1FL	S1JO	-0.083	0.895
S1j	S1JO	-0.162	0.795
Ig	S1DZ	-0.636	0.249
cg2	S1DZ	0.502	0.389
cg1	S1DZ	0.676	0.210
M2	S1DZ	0.708	0.181
M1	S1DZ	0.801	0.103
S1FL	S1DZ	0.527	0.362
S1j	S1DZ	0.445	0.452
S1JO	S1DZ	0.748	0.146
Ig	S1ULp	-0.828	0.084
cg2	S1ULp	0.224	0.717
cg1	S1ULp	0.988	**0.002**
M2	S1ULp	0.987	**0.002**
M1	S1ULp	0.893	**0.041**
S1FL	S1ULp	0.879	**<0.050**
S1j	S1ULp	0.794	0.109
S1JO	S1ULp	0.277	0.652
S1DZ	S1ULp	0.706	0.183
Ig	GI	-0.606	0.279
cg2	GI	0.486	0.407
cg1	GI	0.974	**0.005**
M2	GI	0.953	**0.012**
M1	GI	0.951	**0.013**
S1FL	GI	0.647	0.238
S1j	GI	0.526	0.363
S1JO	GI	0.428	0.472
S1DZ	GI	0.643	0.242
S1ULp	GI	0.928	**0.023**
Ig	DI	-0.862	0.060
cg2	DI	0.327	0.591
cg1	DI	0.821	0.089
M2	DI	0.833	0.080
M1	DI	0.810	0.097
S1FL	DI	0.777	0.122
S1j	DI	0.697	0.191
S1JO	DI	0.484	0.409
S1DZ	DI	0.935	**0.020**
S1ULp	DI	0.868	0.057
GI	DI	0.736	0.156
Ig	AID	-0.716	0.174
cg2	AID	0.522	0.367
cg1	AID	0.688	0.200
M2	AID	0.695	0.193
M1	AID	0.765	0.132
S1FL	AID	0.555	0.332
S1j	AID	0.461	0.435
S1JO	AID	0.666	0.220
S1DZ	AID	0.985	**0.002**
S1ULp	AID	0.718	0.172
GI	AID	0.642	0.242
DI	AID	0.954	**0.012**
Ig	AIV	-0.374	0.535
cg2	AIV	-0.092	0.882
cg1	AIV	0.556	0.331
M2	AIV	0.681	0.206
M1	AIV	0.681	0.206
S1FL	AIV	0.596	0.289
S1j	AIV	0.624	0.260
S1JO	AIV	0.511	0.379
S1DZ	AIV	0.665	0.221
S1ULp	AIV	0.616	0.268
GI	AIV	0.496	0.396

DI	AIV	0.636	0.249
AID	AIV	0.552	0.335
Ig	CI	-0.546	0.342
cg2	CI	0.497	0.394
cg1	CI	0.213	0.731
M2	CI	0.100	0.873
M1	CI	0.109	0.862
S1FL	CI	0.154	0.804
S1j	CI	0.042	0.947
S1JO	CI	0.078	0.900
S1DZ	CI	0.410	0.494
S1ULp	CI	0.215	0.728
GI	CI	0.173	0.781
DI	CI	0.464	0.431
AID	CI	0.553	0.334
AIV	CI	-0.365	0.546
Ig	Pir	-0.334	0.582
cg2	Pir	0.778	0.122
cg1	Pir	0.430	0.470
M2	Pir	0.431	0.469
M1	Pir	0.646	0.239
S1FL	Pir	0.119	0.849
S1j	Pir	0.010	0.987
S1JO	Pir	0.880	**0.049**
S1DZ	Pir	0.895	**0.040**
S1ULp	Pir	0.414	0.489
GI	Pir	0.475	0.418
DI	Pir	0.717	0.173
AID	Pir	0.888	**0.044**
AIV	Pir	0.373	0.537
CI	Pir	0.519	0.370
Ig	Den	-0.436	0.462
cg2	Den	0.317	0.603
cg1	Den	0.067	0.914
M2	Den	-0.071	0.909
M1	Den	-0.131	0.834
S1FL	Den	0.071	0.909
S1j	Den	-0.024	0.969
S1JO	Den	-0.252	0.682
S1DZ	Den	0.072	0.909
S1ULp	Den	0.061	0.923
GI	Den	0.025	0.968
DI	Den	0.197	0.751
AID	Den	0.240	0.698
AIV	Den	-0.622	0.263
CI	Den	0.932	**0.021**
Pir	Den	0.187	0.764
Ig	LSS	-0.785	0.116
cg2	LSS	0.212	0.733
cg1	LSS	0.388	0.519
M2	LSS	0.285	0.642
M1	LSS	0.160	0.797
S1FL	LSS	0.484	0.409
S1j	LSS	0.396	0.509
S1JO	LSS	-0.171	0.783
S1DZ	LSS	0.374	0.535
S1ULp	LSS	0.424	0.477
GI	LSS	0.280	0.648
DI	LSS	0.561	0.325
AID	LSS	0.527	0.362
AIV	LSS	-0.234	0.704
CI	LSS	0.914	**0.030**
Pir	LSS	0.308	0.614
Den	LSS	0.898	**0.039**
Ig	CPDM	-0.510	0.380
cg2	CPDM	0.714	0.175
cg1	CPDM	0.450	0.447
M2	CPDM	0.321	0.599
M1	CPDM	0.365	0.545
S1FL	CPDM	0.173	0.780
S1j	CPDM	0.015	0.981
S1JO	CPDM	0.200	0.748
S1DZ	CPDM	0.428	0.472
S1ULp	CPDM	0.398	0.507
GI	CPDM	0.477	0.417
DI	CPDM	0.485	0.407
AID	CPDM	0.556	0.330
AIV	CPDM	-0.327	0.591
CI	CPDM	0.906	**0.034**
Pir	CPDM	0.554	0.333
Den	CPDM	0.845	0.071
LSS	CPDM	0.815	0.093
Ig	CPDL	-0.850	0.068
cg2	CPDL	0.447	0.450
cg1	CPDL	0.846	0.071
M2	CPDL	0.762	0.134
M1	CPDL	0.684	0.203
S1FL	CPDL	0.687	0.200
S1j	CPDL	0.552	0.334
S1JO	CPDL	0.143	0.819
S1DZ	CPDL	0.604	0.281
S1ULp	CPDL	0.833	0.080
GI	CPDL	0.801	0.103
DI	CPDL	0.782	0.118

AID	CPDL	0.702	0.186
AIV	CPDL	0.128	0.838
CI	CPDL	0.678	0.208
Pir	CPDL	0.468	0.426
Den	CPDL	0.589	0.296
LSS	CPDL	0.797	0.107
CPDM	CPDL	0.820	0.089
Ig	CPVL	-0.404	0.500
cg2	CPVL	0.705	0.184
cg1	CPVL	0.554	0.332
M2	CPVL	0.418	0.483
M1	CPVL	0.446	0.451
S1FL	CPVL	0.173	0.781
S1j	CPVL	0.006	0.992
S1JO	CPVL	0.124	0.843
S1DZ	CPVL	0.261	0.671
S1ULp	CPVL	0.463	0.432
GI	CPVL	0.624	0.261
DI	CPVL	0.355	0.557
AID	CPVL	0.372	0.537
AIV	CPVL	-0.337	0.579
CI	CPVL	0.684	0.203
Pir	CPVL	0.367	0.543
Den	CPVL	0.692	0.195
LSS	CPVL	0.638	0.246
CPDM	CPVL	0.921	**0.026**
CPDL	CPVL	0.815	0.093
Ig	CPVM	-0.465	0.430
cg2	CPVM	0.493	0.398
cg1	CPVM	0.779	0.120
M2	CPVM	0.681	0.205
M1	CPVM	0.638	0.247
S1FL	CPVM	0.424	0.476
S1j	CPVM	0.282	0.646
S1JO	CPVM	0.057	0.927
S1DZ	CPVM	0.234	0.705
S1ULp	CPVM	0.694	0.194
GI	CPVM	0.835	0.079
DI	CPVM	0.398	0.507
AID	CPVM	0.301	0.623
AIV	CPVM	-0.045	0.943
CI	CPVM	0.331	0.586
Pir	CPVM	0.177	0.775
Den	CPVM	0.361	0.550
LSS	CPVM	0.427	0.474
CPDM	CPVM	0.667	0.219
CPDL	CPVM	0.814	0.094
CPVL	CPVM	0.880	**0.049**
Ig	AcCo	-0.650	0.235
cg2	AcCo	0.244	0.693
cg1	AcCo	0.651	0.234
M2	AcCo	0.530	0.358
M1	AcCo	0.375	0.534
S1FL	AcCo	0.532	0.356
S1j	AcCo	0.416	0.486
S1JO	AcCo	-0.281	0.647
S1DZ	AcCo	0.123	0.844
S1ULp	AcCo	0.613	0.271
GI	AcCo	0.622	0.263
DI	AcCo	0.386	0.521
AID	AcCo	0.249	0.686
AIV	AcCo	-0.234	0.705
CI	AcCo	0.551	0.335
Pir	AcCo	0.013	0.983
Den	AcCo	0.652	0.233
LSS	AcCo	0.726	0.165
CPDM	AcCo	0.734	0.158
CPDL	AcCo	0.864	0.059
CPVL	AcCo	0.849	0.069
CPVM	AcCo	0.887	**0.045**
Ig	AcSh	-0.947	**0.014**
cg2	AcSh	-0.258	0.675
cg1	AcSh	0.633	0.252
M2	AcSh	0.605	0.280
M1	AcSh	0.340	0.576
S1FL	AcSh	0.914	**0.030**
S1j	AcSh	0.893	**0.041**
S1JO	AcSh	-0.329	0.588
S1DZ	AcSh	0.367	0.544
S1ULp	AcSh	0.716	0.173
GI	AcSh	0.454	0.443
DI	AcSh	0.666	0.220
AID	AcSh	0.458	0.438
AIV	AcSh	0.251	0.684
CI	AcSh	0.425	0.476
Pir	AcSh	0.017	0.978
Den	AcSh	0.422	0.479
LSS	AcSh	0.746	0.148
CPDM	AcSh	0.360	0.552
CPDL	AcSh	0.736	0.157
CPVL	AcSh	0.298	0.626
CPVM	AcSh	0.411	0.492
AcCo	AcSh	0.677	0.209
Ig	LSD	-0.833	0.080

cg2	LSD	0.116	0.853
cg1	LSD	0.507	0.384
M2	LSD	0.527	0.362
M1	LSD	0.474	0.420
S1FL	LSD	0.676	0.210
S1j	LSD	0.642	0.242
S1JO	LSD	0.320	0.600
S1DZ	LSD	0.840	0.075
S1ULp	LSD	0.602	0.283
GI	LSD	0.364	0.547
DI	LSD	0.896	**0.040**
AID	LSD	0.881	**0.048**
AIV	LSD	0.501	0.390
CI	LSD	0.572	0.314
Pir	LSD	0.640	0.244
Den	LSD	0.323	0.596
LSS	LSD	0.654	0.231
CPDM	LSD	0.401	0.504
CPDL	LSD	0.599	0.286
CPVL	LSD	0.129	0.837
CPVM	LSD	0.044	0.944
AcCo	LSD	0.198	0.750
AcSh	LSD	0.678	0.209
Ig	LSI	-0.972	**0.006**
cg2	LSI	0.168	0.787
cg1	LSI	0.694	0.194
M2	LSI	0.657	0.228
M1	LSI	0.534	0.354
S1FL	LSI	0.798	0.105
S1j	LSI	0.724	0.167
S1JO	LSI	0.104	0.868
S1DZ	LSI	0.719	0.171
S1ULp	LSI	0.758	0.137
GI	LSI	0.559	0.328
DI	LSI	0.891	**0.043**
AID	LSI	0.809	0.097
AIV	LSI	0.302	0.622
CI	LSI	0.699	0.189
Pir	LSI	0.497	0.394
Den	LSI	0.549	0.338
LSS	LSI	0.856	0.064
CPDM	LSI	0.634	0.251
CPDL	LSI	0.863	0.060
CPVL	LSI	0.465	0.430
CPVM	LSI	0.420	0.481
AcCo	LSI	0.606	0.278
AcSh	LSI	0.868	0.056

LSD	LSI	0.899	**0.038**
Ig	LSV	-0.959	**0.010**
cg2	LSV	0.008	0.990
cg1	LSV	0.571	0.315
M2	LSV	0.544	0.344
M1	LSV	0.386	0.521
S1FL	LSV	0.790	0.112
S1j	LSV	0.745	0.149
S1JO	LSV	-0.017	0.978
S1DZ	LSV	0.638	0.247
S1ULp	LSV	0.659	0.226
GI	LSV	0.405	0.498
DI	LSV	0.825	0.086
AID	LSV	0.732	0.160
AIV	LSV	0.271	0.659
CI	LSV	0.676	0.210
Pir	LSV	0.395	0.511
Den	LSV	0.551	0.336
LSS	LSV	0.857	0.063
CPDM	LSV	0.537	0.351
CPDL	LSV	0.764	0.132
CPVL	LSV	0.331	0.587
CPVM	LSV	0.273	0.657
AcCo	LSV	0.529	0.359
AcSh	LSV	0.895	**0.040**
LSD	LSV	0.911	**0.032**
LSI	LSV	0.981	**0.003**
Ig	Shi	-0.928	**0.023**
cg2	Shi	-0.039	0.951
cg1	Shi	0.844	0.072
M2	Shi	0.806	0.100
M1	Shi	0.587	0.298
S1FL	Shi	0.930	**0.022**
S1j	Shi	0.867	0.057
S1JO	Shi	-0.166	0.789
S1DZ	Shi	0.439	0.460
S1ULp	Shi	0.883	0.047
GI	Shi	0.723	0.168
DI	Shi	0.724	0.166
AID	Shi	0.514	0.375
AIV	Shi	0.317	0.604
CI	Shi	0.366	0.545
Pir	Shi	0.109	0.861
Den	Shi	0.345	0.570
LSS	Shi	0.667	0.219
CPDM	Shi	0.453	0.443
CPDL	Shi	0.863	0.059

CPVL	Shi	0.495	0.396
CPVM	Shi	0.678	0.208
AcCo	Shi	0.805	0.101
AcSh	Shi	0.936	**0.019**
LSD	Shi	0.576	0.309
LSI	Shi	0.835	0.078
LSV	Shi	0.796	0.107
Ig	MS	-0.868	0.056
cg2	MS	0.143	0.819
cg1	MS	0.929	**0.022**
M2	MS	0.953	**0.012**
M1	MS	0.858	0.063
S1FL	MS	0.912	**0.031**
S1j	MS	0.850	0.068
S1JO	MS	0.320	0.600
S1DZ	MS	0.794	0.109
S1ULp	MS	0.973	**0.005**
GI	MS	0.838	0.077
DI	MS	0.934	**0.020**
AID	MS	0.796	0.107
AIV	MS	0.720	0.170
CI	MS	0.222	0.719
Pir	MS	0.475	0.418
Den	MS	0.022	0.972
LSS	MS	0.430	0.470
CPDM	MS	0.320	0.599
CPDL	MS	0.770	0.128
CPVL	MS	0.310	0.612
CPVM	MS	0.515	0.374
AcCo	MS	0.473	0.421
AcSh	MS	0.744	0.150
LSD	MS	0.744	0.149
LSI	MS	0.813	0.094
LSV	MS	0.743	0.150
Shi	MS	0.848	0.069
Ig	VDB	-0.042	0.947
cg2	VDB	-0.157	0.800
cg1	VDB	0.539	0.348
M2	VDB	0.588	0.297
M1	VDB	0.510	0.380
S1FL	VDB	0.395	0.510
S1j	VDB	0.398	0.507
S1JO	VDB	0.005	0.993
S1DZ	VDB	-0.037	0.953
S1ULp	VDB	0.500	0.391
GI	VDB	0.575	0.310
DI	VDB	0.070	0.912

AID	VDB	-0.123	0.844
AIV	VDB	0.501	0.390
CI	VDB	-0.660	0.226
Pir	VDB	-0.276	0.654
Den	VDB	-0.621	0.263
LSS	VDB	-0.445	0.452
CPDM	VDB	-0.347	0.567
CPDL	VDB	0.092	0.884
CPVL	VDB	-0.012	0.984
CPVM	VDB	0.452	0.445
AcCo	VDB	0.186	0.765
AcSh	VDB	0.100	0.873
LSD	VDB	-0.292	0.633
LSI	VDB	-0.150	0.810
LSV	VDB	-0.233	0.706
Shi	VDB	0.339	0.577
MS	VDB	0.392	0.514
Ig	Icj	-0.808	0.098
cg2	Icj	-0.045	0.942
cg1	Icj	0.925	**0.024**
M2	Icj	0.947	**0.015**
M1	Icj	0.785	0.116
S1FL	Icj	0.947	**0.015**
S1j	Icj	0.904	**0.035**
S1JO	Icj	0.091	0.885
S1DZ	Icj	0.562	0.324
S1ULp	Icj	0.961	**0.009**
GI	Icj	0.831	0.081
DI	Icj	0.772	0.126
AID	Icj	0.562	0.324
AIV	Icj	0.670	0.216
CI	Icj	0.030	0.962
Pir	Icj	0.188	0.762
Den	Icj	-0.075	0.905
LSS	Icj	0.322	0.597
CPDM	Icj	0.178	0.774
CPDL	Icj	0.707	0.182
CPVL	Icj	0.272	0.658
CPVM	Icj	0.588	0.297
AcCo	Icj	0.549	0.338
AcSh	Icj	0.771	0.127
LSD	Icj	0.536	0.351
LSI	Icj	0.685	0.201
LSV	Icj	0.622	0.262
Shi	Icj	0.898	**0.039**
MS	Icj	0.947	**0.015**
VDB	Icj	0.610	0.275

Ig	VP	-0.897	**0.039**
cg2	VP	-0.333	0.584
cg1	VP	0.527	0.361
M2	VP	0.489	0.404
M1	VP	0.202	0.744
S1FL	VP	0.853	0.066
S1j	VP	0.842	0.073
S1JO	VP	-0.459	0.437
S1DZ	VP	0.237	0.701
S1ULp	VP	0.613	0.272
GI	VP	0.342	0.573
DI	VP	0.554	0.332
AID	VP	0.342	0.574
AIV	VP	0.126	0.839
CI	VP	0.432	0.467
Pir	VP	-0.097	0.877
Den	VP	0.478	0.415
LSS	VP	0.760	0.136
CPDM	VP	0.341	0.574
CPDL	VP	0.678	0.208
CPVL	VP	0.278	0.650
CPVM	VP	0.369	0.541
AcCo	VP	0.683	0.204
AcSh	VP	0.988	**0.001**
LSD	VP	0.605	0.280
LSI	VP	0.815	0.093
LSV	VP	0.860	0.062
Shi	VP	0.895	**0.040**
MS	VP	0.635	0.250
VDB	VP	0.041	0.947
Icj	VP	0.683	0.204
Ig	Tu	-0.500	0.391

cg2	Tu	0.386	0.521
cg1	Tu	0.878	0.050
M2	Tu	0.927	**0.023**
M1	Tu	0.978	**0.004**
S1FL	Tu	0.616	0.268
S1j	Tu	0.541	0.346
S1JO	Tu	0.653	0.232
S1DZ	Tu	0.787	0.114
S1ULp	Tu	0.865	0.058
GI	Tu	0.893	**0.042**
DI	Tu	0.782	0.118
AID	Tu	0.722	0.169
AIV	Tu	0.811	0.096
CI	Tu	-0.058	0.926
Pir	Tu	0.589	0.296
Den	Tu	-0.311	0.611
LSS	Tu	0.016	0.979
CPDM	Tu	0.173	0.781
CPDL	Tu	0.552	0.335
CPVL	Tu	0.255	0.679
CPVM	Tu	0.502	0.389
AcCo	Tu	0.223	0.719
AcSh	Tu	0.304	0.619
LSD	Tu	0.462	0.433
LSI	Tu	0.460	0.435
LSV	Tu	0.331	0.587
Shi	Tu	0.530	0.358
MS	Tu	0.857	0.064
VDB	Tu	0.581	0.304
Icj	Tu	0.797	0.106
VP	Tu	0.161	0.797

Supplementary Table S4. Results of Pearson's *r* correlations in KET/ASE group. Correlation coefficients and p-values for all possible pairs are reported, significant values were given in bold.

ROI	ROI	Pearson's *r*	p-value
Ig	cg2	0.366	0.545
Ig	cg1	0.056	0.929
cg2	cg1	0.910	**0.032**
Ig	M2	0.359	0.553
cg2	M2	0.930	**0.022**
cg1	M2	0.854	0.066
Ig	M1	0.657	0.228
cg2	M1	0.813	0.094
cg1	M1	0.648	0.237
M2	M1	0.924	**0.025**
Ig	S1FL	0.338	0.578
cg2	S1FL	0.848	0.069
cg1	S1FL	0.708	0.181
M2	S1FL	0.960	**0.010**
M1	S1FL	0.897	**0.039**
Ig	S1j	0.450	0.446
cg2	S1j	0.927	**0.023**
cg1	S1j	0.833	0.080
M2	S1j	0.994	**0.001**
M1	S1j	0.954	**0.012**
S1FL	S1j	0.943	**0.016**
Ig	S1JO	0.871	0.055
cg2	S1JO	0.675	0.211
cg1	S1JO	0.478	0.416
M2	S1JO	0.732	0.160
M1	S1JO	0.918	**0.028**
S1FL	S1JO	0.658	0.227
S1j	S1JO	0.800	0.104
cg2	DI	0.833	0.080
cg1	DI	0.573	0.313
M2	DI	0.840	0.075
M1	DI	0.939	**0.018**
S1FL	DI	0.823	0.087
S1j	DI	0.881	**0.048**
S1JO	DI	0.922	**0.026**
S1DZ	DI	0.924	**0.025**
S1ULp	DI	0.954	**0.012**
GI	DI	0.965	**0.008**
Ig	AID	0.713	0.176
cg2	AID	0.899	**0.038**
cg1	AID	0.696	0.192
M2	AID	0.898	**0.039**
M1	AID	0.954	**0.012**
S1FL	AID	0.847	0.070
Ig	S1DZ	0.527	0.362
cg2	S1DZ	0.974	**0.005**
cg1	S1DZ	0.840	0.075
M2	S1DZ	0.959	**0.010**
M1	S1DZ	0.922	**0.026**
S1FL	S1DZ	0.891	**0.042**
S1j	S1DZ	0.973	**0.005**
S1JO	S1DZ	0.815	0.092
Ig	S1ULp	0.576	0.309
cg2	S1ULp	0.931	**0.021**
cg1	S1ULp	0.758	0.138
M2	S1ULp	0.962	**0.009**
M1	S1ULp	0.961	**0.009**
S1FL	S1ULp	0.934	**0.020**
S1j	S1ULp	0.978	**0.004**
S1JO	S1ULp	0.844	0.072
S1DZ	S1ULp	0.986	**0.002**
Ig	GI	0.603	0.281
cg2	GI	0.940	**0.018**
cg1	GI	0.734	0.158
M2	GI	0.927	**0.024**
M1	GI	0.925	**0.024**
S1FL	GI	0.903	**0.036**
S1j	GI	0.944	**0.016**
S1JO	GI	0.825	0.086
S1DZ	GI	0.982	**0.003**
S1ULp	GI	0.990	**0.001**
Ig	DI	0.787	0.114
S1DZ	AIV	0.883	**0.047**
S1ULp	AIV	0.932	**0.021**
GI	AIV	0.894	**0.041**
DI	AIV	0.935	**0.020**
AID	AIV	0.938	**0.018**
Ig	CI	0.824	0.086
cg2	CI	0.619	0.265
cg1	CI	0.474	0.420
M2	CI	0.710	0.179
M1	CI	0.901	**0.037**
S1FL	CI	0.618	0.267
S1j	CI	0.780	0.120
S1JO	CI	0.987	**0.002**
S1DZ	CI	0.769	0.129
S1ULp	CI	0.795	0.108
GI	CI	0.757	0.139

S1j	AID	0.934	**0.020**
S1JO	AID	0.916	**0.029**
S1DZ	AID	0.970	**0.006**
S1ULp	AID	0.979	**0.004**
GI	AID	0.981	**0.003**
DI	AID	0.986	**0.002**
Ig	AIV	0.711	0.178
cg2	AIV	0.755	0.140
cg1	AIV	0.575	0.311
M2	AIV	0.880	**0.049**
M1	AIV	0.995	**<0.001**
S1FL	AIV	0.858	0.063
S1j	AIV	0.919	**0.027**
S1JO	AIV	0.942	**0.017**
DI	Pir	0.691	0.196
AID	Pir	0.780	0.120
AIV	Pir	0.846	0.071
CI	Pir	0.798	0.106
Ig	Den	0.462	0.434
cg2	Den	0.793	0.110
cg1	Den	0.807	0.098
M2	Den	0.879	**<0.050**
M1	Den	0.889	**0.044**
S1FL	Den	0.740	0.153
S1j	Den	0.908	**0.033**
S1JO	Den	0.834	0.079
S1DZ	Den	0.862	0.060
S1ULp	Den	0.842	0.074
GI	Den	0.778	0.121
DI	Den	0.755	0.140
AID	Den	0.837	0.077
AIV	Den	0.872	0.054
CI	Den	0.871	0.055
Pir	Den	0.984	**0.002**
Ig	LSS	0.727	0.164
cg2	LSS	0.403	0.501
cg1	LSS	0.360	0.552
M2	LSS	0.319	0.601
M1	LSS	0.501	0.390
S1FL	LSS	0.110	0.860
S1j	LSS	0.409	0.494
S1JO	LSS	0.764	0.132
S1DZ	LSS	0.489	0.403
S1ULp	LSS	0.433	0.466
S1FL	CPDL	0.553	0.334
S1j	CPDL	0.781	0.119
S1JO	CPDL	0.920	**0.027**
S1DZ	CPDL	0.841	0.075
S1ULp	CPDL	0.802	0.103

DI	CI	0.858	0.063
AID	CI	0.865	0.058
AIV	CI	0.929	**0.022**
Ig	Pir	0.331	0.586
cg2	Pir	0.771	0.127
cg1	Pir	0.820	0.089
M2	Pir	0.898	**0.038**
M1	Pir	0.869	0.056
S1FL	Pir	0.781	0.119
S1j	Pir	0.911	**0.031**
S1JO	Pir	0.752	0.143
S1DZ	Pir	0.832	0.081
S1ULp	Pir	0.820	0.089
GI	Pir	0.742	0.151
GI	LSS	0.432	0.468
DI	LSS	0.549	0.338
AID	LSS	0.582	0.304
AIV	LSS	0.532	0.356
CI	LSS	0.788	0.113
Pir	LSS	0.511	0.379
Den	LSS	0.650	0.236
Ig	CPDM	0.661	0.224
cg2	CPDM	0.908	**0.033**
cg1	CPDM	0.782	0.118
M2	CPDM	0.889	**0.044**
M1	CPDM	0.924	**0.025**
S1FL	CPDM	0.778	0.122
S1j	CPDM	0.929	**0.023**
S1JO	CPDM	0.912	**0.031**
S1DZ	CPDM	0.965	**0.008**
S1ULp	CPDM	0.944	**0.016**
GI	CPDM	0.935	**0.020**
DI	CPDM	0.930	**0.022**
AID	CPDM	0.974	**0.005**
AIV	CPDM	0.904	**0.035**
CI	CPDM	0.885	**0.046**
Pir	CPDM	0.849	0.069
Den	CPDM	0.912	**0.031**
LSS	CPDM	0.696	0.192
Ig	CPDL	0.740	0.153
cg2	CPDL	0.769	0.129
cg1	CPDL	0.669	0.217
M2	CPDL	0.717	0.173
M1	CPDL	0.815	0.093
Den	CPVL	0.781	0.119
LSS	CPVL	0.848	0.069
CPDM	CPVL	0.903	**0.036**
CPDL	CPVL	0.951	**0.013**
Ig	CPVM	0.927	**0.024**

GI	CPDL	0.796	0.107
DI	CPDL	0.841	0.074
AID	CPDL	0.885	**0.046**
AIV	CPDL	0.814	0.093
CI	CPDL	0.911	**0.031**
Pir	CPDL	0.771	0.127
Den	CPDL	0.872	0.054
LSS	CPDL	0.883	**0.047**
CPDM	CPDL	0.951	**0.013**
Ig	CPVL	0.904	**0.035**
cg2	CPVL	0.657	0.228
cg1	CPVL	0.456	0.440
M2	CPVL	0.650	0.235
M1	CPVL	0.843	0.073
S1FL	CPVL	0.549	0.338
S1j	CPVL	0.728	0.164
S1JO	CPVL	0.982	**0.003**
S1DZ	CPVL	0.782	0.118
S1ULp	CPVL	0.789	0.112
GI	CPVL	0.790	0.112
DI	CPVL	0.897	**0.039**
AID	CPVL	0.891	**0.043**
AIV	CPVL	0.867	0.057
CI	CPVL	0.959	**0.010**
Pir	CPVL	0.671	0.215
S1FL	AcCo	0.115	0.853
S1j	AcCo	0.313	0.608
S1JO	AcCo	0.816	0.092
S1DZ	AcCo	0.378	0.530
S1ULp	AcCo	0.400	0.504
GI	AcCo	0.408	0.496
DI	AcCo	0.612	0.272
AID	AcCo	0.565	0.321
AIV	AcCo	0.592	0.293
CI	AcCo	0.815	0.092
Pir	AcCo	0.330	0.588
Den	AcCo	0.472	0.422
LSS	AcCo	0.872	0.054
CPDM	AcCo	0.582	0.303
CPDL	AcCo	0.750	0.144
CPVL	AcCo	0.872	0.054
CPVM	AcCo	0.933	**0.021**
Ig	AcSh	0.838	0.077
cg2	AcSh	-0.055	0.931
cg1	AcSh	-0.219	0.724
M2	AcSh	-0.062	0.922
M1	AcSh	0.284	0.643
S1FL	AcSh	-0.152	0.807
S1j	AcSh	0.047	0.940

cg2	CPVM	0.540	0.347
cg1	CPVM	0.347	0.567
M2	CPVM	0.542	0.346
M1	CPVM	0.776	0.123
S1FL	CPVM	0.434	0.465
S1j	CPVM	0.630	0.255
S1JO	CPVM	0.962	**0.009**
S1DZ	CPVM	0.682	0.205
S1ULp	CPVM	0.692	0.195
GI	CPVM	0.689	0.198
DI	CPVM	0.827	0.084
AID	CPVM	0.814	0.094
AIV	CPVM	0.813	0.094
CI	CPVM	0.951	**0.013**
Pir	CPVM	0.610	0.275
Den	CPVM	0.730	0.162
LSS	CPVM	0.887	**0.045**
CPDM	CPVM	0.834	0.079
CPDL	CPVM	0.922	**0.026**
CPVL	CPVM	0.989	**0.001**
Ig	AcCo	0.937	**0.019**
cg2	AcCo	0.215	0.729
cg1	AcCo	0.012	0.985
M2	AcCo	0.209	0.736
M1	AcCo	0.526	0.363
AIV	AcSh	0.365	0.545
CI	AcSh	0.648	0.237
Pir	AcSh	0.118	0.850
Den	AcSh	0.263	0.669
LSS	AcSh	0.809	0.097
CPDM	AcSh	0.343	0.572
CPDL	AcSh	0.567	0.319
CPVL	AcSh	0.704	0.184
CPVM	AcSh	0.800	0.104
AcCo	AcSh	0.961	**0.009**
Ig	LSD	0.814	0.093
cg2	LSD	0.295	0.630
cg1	LSD	0.210	0.735
M2	LSD	0.297	0.627
M1	LSD	0.554	0.332
S1FL	LSD	0.135	0.829
S1j	LSD	0.395	0.510
S1JO	LSD	0.821	0.088
S1DZ	LSD	0.436	0.463
S1ULp	LSD	0.424	0.477
GI	LSD	0.405	0.499
DI	LSD	0.569	0.317
AID	LSD	0.572	0.313
AIV	LSD	0.606	0.278

S1JO	AcSh	0.631	0.253
S1DZ	AcSh	0.109	0.861
S1ULp	AcSh	0.131	0.834
GI	AcSh	0.139	0.824
DI	AcSh	0.372	0.537
AID	AcSh	0.314	0.607
CPVL	LSD	0.873	0.053
CPVM	LSD	0.930	**0.022**
AcCo	LSD	0.949	**0.014**
AcSh	LSD	0.900	**0.037**
Ig	LSI	0.585	0.301
cg2	LSI	0.254	0.680
cg1	LSI	0.247	0.688
M2	LSI	0.077	0.902
M1	LSI	0.225	0.716
S1FL	LSI	-0.152	0.807
S1j	LSI	0.164	0.792
S1JO	LSI	0.537	0.350
S1DZ	LSI	0.289	0.637
S1ULp	LSI	0.201	0.746
GI	LSI	0.227	0.714
DI	LSI	0.333	0.584
AID	LSI	0.368	0.542
AIV	LSI	0.250	0.685
CI	LSI	0.552	0.335
Pir	LSI	0.265	0.667
Den	LSI	0.420	0.481
LSS	LSI	0.947	**0.015**
CPDM	LSI	0.503	0.387
CPDL	LSI	0.737	0.155
CPVL	LSI	0.671	0.215
CPVM	LSI	0.723	0.168
AcCo	LSI	0.768	0.129
AcSh	LSI	0.761	0.135
LSD	LSI	0.853	0.066
Ig	LSV	0.868	0.056
S1FL	Shi	-0.163	0.794
S1j	Shi	0.082	0.896
S1JO	Shi	0.629	0.256
S1DZ	Shi	0.127	0.839
S1ULp	Shi	0.127	0.839
GI	Shi	0.115	0.853
DI	Shi	0.327	0.591
AID	Shi	0.302	0.622
AIV	Shi	0.363	0.548
CI	Shi	0.673	0.214
Pir	Shi	0.228	0.713
Den	Shi	0.364	0.547
LSS	Shi	0.875	0.052

CI	LSD	0.856	0.064
Pir	LSD	0.510	0.380
Den	LSD	0.638	0.247
LSS	LSD	0.961	**0.009**
CPDM	LSD	0.654	0.231
CPDL	LSD	0.836	0.077
cg2	LSV	0.598	0.287
cg1	LSV	0.353	0.561
M2	LSV	0.716	0.174
M1	LSV	0.927	**0.023**
S1FL	LSV	0.710	0.179
S1j	LSV	0.777	0.122
S1JO	LSV	0.969	**0.007**
S1DZ	LSV	0.764	0.133
S1ULp	LSV	0.831	0.081
GI	LSV	0.807	0.099
DI	LSV	0.918	**0.028**
AID	LSV	0.883	**0.047**
AIV	LSV	0.959	**0.010**
CI	LSV	0.949	**0.014**
Pir	LSV	0.693	0.194
Den	LSV	0.753	0.142
LSS	LSV	0.604	0.281
CPDM	LSV	0.831	0.081
CPDL	LSV	0.796	0.107
CPVL	LSV	0.917	**0.028**
CPVM	LSV	0.896	**0.040**
AcCo	LSV	0.762	0.134
AcSh	LSV	0.579	0.306
LSD	LSV	0.716	0.174
LSI	LSV	0.341	0.574
Ig	Shi	0.758	0.138
cg2	Shi	-0.025	0.968
cg1	Shi	-0.108	0.863
M2	Shi	-0.024	0.969
M1	Shi	0.289	0.637
S1DZ	MS	0.429	0.471
S1ULp	MS	0.275	0.655
GI	MS	0.298	0.627
DI	MS	0.204	0.742
AID	MS	0.327	0.592
AIV	MS	0.102	0.870
CI	MS	0.243	0.694
Pir	MS	0.392	0.514
Den	MS	0.469	0.425
LSS	MS	0.616	0.268
CPDM	MS	0.500	0.391
CPDL	MS	0.598	0.286
CPVL	MS	0.347	0.567

CPDM	Shi	0.376	0.533
CPDL	Shi	0.617	0.267
CPVL	Shi	0.701	0.188
CPVM	Shi	0.798	0.105
AcCo	Shi	0.936	**0.019**
AcSh	Shi	0.977	**0.004**
LSD	Shi	0.944	**0.016**
LSI	Shi	0.827	0.084
LSV	Shi	0.543	0.345
Ig	MS	0.042	0.947
cg2	MS	0.540	0.348
cg1	MS	0.699	0.189
M2	MS	0.299	0.625
M1	MS	0.153	0.806
S1FL	MS	0.044	0.944
S1j	MS	0.316	0.605
S1JO	MS	0.237	0.702
GI	VDB	0.261	0.671
DI	VDB	0.453	0.443
AID	VDB	0.410	0.493
AIV	VDB	0.342	0.574
CI	VDB	0.615	0.269
Pir	VDB	0.102	0.871
Den	VDB	0.273	0.657
LSS	VDB	0.872	0.054
CPDM	VDB	0.450	0.447
CPDL	VDB	0.667	0.218
CPVL	VDB	0.752	0.143
CPVM	VDB	0.820	0.089
AcCo	VDB	0.937	**0.019**
AcSh	VDB	0.941	**0.017**
LSD	VDB	0.879	**<0.050**
LSI	VDB	0.880	**0.049**
LSV	VDB	0.532	0.356
Shi	VDB	0.913	**0.030**
MS	VDB	0.335	0.582
Ig	Icj	0.878	**<0.050**
cg2	Icj	0.130	0.835
cg1	Icj	-0.023	0.971
M2	Icj	0.168	0.787
M1	Icj	0.494	0.398
S1FL	Icj	0.066	0.916
S1j	Icj	0.273	0.657
S1JO	Icj	0.785	0.116
S1DZ	Icj	0.306	0.617
S1ULp	Icj	0.331	0.586
GI	Icj	0.317	0.603
DI	VP	0.422	0.479
AID	VP	0.386	0.521

CPVM	MS	0.314	0.606
AcCo	MS	0.176	0.777
AcSh	MS	0.095	0.879
LSD	MS	0.383	0.524
LSI	MS	0.713	0.176
LSV	MS	-0.005	0.994
Shi	MS	0.227	0.714
Ig	VDB	0.845	0.072
cg2	VDB	0.127	0.838
cg1	VDB	-0.055	0.930
M2	VDB	0.002	0.997
M1	VDB	0.284	0.643
S1FL	VDB	-0.122	0.845
S1j	VDB	0.107	0.864
S1JO	VDB	0.635	0.249
S1DZ	VDB	0.236	0.703
S1ULp	VDB	0.217	0.726
DI	Icj	0.529	0.360
AID	Icj	0.490	0.402
AIV	Icj	0.566	0.320
CI	Icj	0.811	0.096
Pir	Icj	0.357	0.555
Den	Icj	0.485	0.407
LSS	Icj	0.866	0.057
CPDM	Icj	0.526	0.362
CPDL	Icj	0.710	0.179
CPVL	Icj	0.827	0.084
CPVM	Icj	0.902	**0.036**
AcCo	Icj	0.985	**0.002**
AcSh	Icj	0.969	**0.007**
LSD	Icj	0.964	**0.008**
LSI	Icj	0.750	0.144
LSV	Icj	0.733	0.159
Shi	Icj	0.968	**0.007**
MS	Icj	0.142	0.820
VDB	Icj	0.897	**0.039**
Ig	VP	0.768	0.129
cg2	VP	0.005	0.994
cg1	VP	-0.097	0.877
M2	VP	0.144	0.817
M1	VP	0.477	0.417
S1FL	VP	0.066	0.915
S1j	VP	0.240	0.698
S1JO	VP	0.723	0.168
S1DZ	VP	0.206	0.739
S1ULp	VP	0.258	0.676
GI	VP	0.208	0.737
GI	Tu	0.559	0.327
DI	Tu	0.753	0.142

AIV	VP	0.557	0.329
CI	VP	0.784	0.117
Pir	VP	0.395	0.510
Den	VP	0.484	0.408
LSS	VP	0.748	0.146
CPDM	VP	0.421	0.480
CPDL	VP	0.589	0.296
CPVL	VP	0.720	0.170
CPVM	VP	0.807	0.099
AcCo	VP	0.899	**0.038**
AcSh	VP	0.904	**0.035**
LSD	VP	0.899	**0.038**
LSI	VP	0.588	0.297
LSV	VP	0.711	0.178
Shi	VP	0.921	**0.026**
MS	VP	-0.030	0.962
VDB	VP	0.742	0.151
Icj	VP	0.957	**0.011**
Ig	Tu	0.980	**0.003**
cg2	Tu	0.320	0.600
cg1	Tu	0.057	0.927
M2	Tu	0.370	0.540
M1	Tu	0.684	0.202
S1FL	Tu	0.334	0.583
S1j	Tu	0.464	0.431
S1JO	Tu	0.897	**0.039**
S1DZ	Tu	0.503	0.388
S1ULp	Tu	0.558	0.328

AID	Tu	0.690	0.197
AIV	Tu	0.746	0.147
CI	Tu	0.881	0.048
Pir	Tu	0.418	0.483
Den	Tu	0.536	0.352
LSS	Tu	0.761	0.135
CPDM	Tu	0.659	0.226
CPDL	Tu	0.752	0.143
CPVL	Tu	0.911	**0.031**
CPVM	Tu	0.947	**0.015**
AcCo	Tu	0.961	**0.009**
AcSh	Tu	0.873	0.053
LSD	Tu	0.875	0.052
LSI	Tu	0.590	0.295
LSV	Tu	0.896	**0.040**
Shi	Tu	0.821	0.088
MS	Tu	0.019	0.976
VDB	Tu	0.827	0.084
Icj	Tu	0.935	**0.020**
VP	Tu	0.161	0.797

Supplementary Table S5. Results of permutation test to assess significant differences in pairs of edge weight between VEH/VEH and VEH/ASE groups. The p-values for all possible pairs are reported, significant values were given in bold.

**ROI**	**ROI**	**p-value**
M1	GI	**0.01**
S1j	AIV	**0.01**
S1FL	CPDL	**0.01**
S1j	CPDL	**0.01**
S1FL	CPVL	**0.01**
S1j	CPVL	**0.01**
S1FL	CI	**0.02**
S1j	CI	**0.02**
M2	Den	**0.02**
S1FL	Den	**0.02**
AIV	CPDL	**0.02**
AIV	CPVL	**0.02**
M1	CPVM	**0.02**
S1j	AcSh	**0.02**
S1FL	AIV	**0.03**
S1FL	LSS	**0.03**
M1	CPVL	**0.03**
GI	Icj	**0.04**
S1j	Den	**<0.05**
cg1	CPDL	**<0.05**
M1	CPDL	**<0.05**
cg1	CPVL	**<0.05**
M2	MS	**<0.05**
cg1	LSS	0.06
VDB	VP	0.06
CI	Pir	0.07
S1j	LSS	0.07
Pir	CPDM	0.07
cg1	MS	0.07
M1	Den	0.08
AIV	Den	0.08
cg2	CPDM	0.08
CI	CPVM	0.08
LSD	LSV	0.08
cg1	AIV	0.09
M1	AIV	0.09
M1	CPDM	0.1
LSS	CPDM	0.1
AID	CPVL	0.1
AID	CPDL	0.11
M2	Shi	0.11
Ig	cg2	0.12
M1	LSS	0.12
S1j	CPDM	0.12
M2	CPDL	0.12
CPDL	AcSh	0.12
DI	Shi	0.12
AIV	MS	0.12
cg2	S1JO	0.13
AIV	CI	0.13
cg1	Den	0.13
M2	CPVL	0.13
LSS	AcCo	0.13
CPDM	AcSh	0.13
S1DZ	Icj	0.13
DI	Icj	0.13
M1	S1DZ	0.14
M1	CI	0.14
M2	LSI	0.14
cg1	Shi	0.14
M2	LSS	0.15
S1FL	AcSh	0.15
MS	VP	0.15
S1ULp	GI	0.16
AIV	CPDM	0.16
cg1	LSI	0.16
M2	LSV	0.16
S1FL	S1j	0.17
S1JO	LSI	0.17
M1	S1FL	0.18
AID	Pir	0.18
AIV	LSS	0.18
S1FL	CPVM	0.18
S1JO	Shi	0.18
cg2	MS	0.18
cg2	Icj	0.18
S1JO	Icj	0.18
S1ULp	Icj	0.18
DI	CPDL	0.19
cg1	M2	0.2
S1JO	CPVL	0.2
S1ULp	Tu	0.2
S1JO	CPVM	0.21
M2	AIV	0.22
cg2	S1DZ	0.23
S1FL	CPDM	0.23
Pir	CPVM	0.23

AcCo	VDB	0.23
LSS	CPDL	0.24
S1j	CPVM	0.24
S1ULp	VP	0.24
AID	Den	0.25
CI	AcSh	0.25
CPVL	AcSh	0.25
CPDL	MS	0.25
cg2	GI	0.26
CI	Den	0.26
Den	LSS	0.27
S1FL	MS	0.29
S1JO	CI	0.3
GI	DI	0.31
GI	CPVL	0.31
S1j	MS	0.31
AcCo	MS	0.31
S1FL	AID	0.32
S1JO	CPDM	0.32
Den	CPDL	0.32
LSS	CPVL	0.32
CPVM	AcSh	0.32
cg2	DI	0.33
CI	CPDM	0.33
CPVL	MS	0.33
M1	S1j	0.34
M1	AcSh	0.36
CPDL	Icj	0.36
MS	Tu	0.36
AcCo	VP	0.37
S1j	AID	0.38
Pir	LSS	0.38
Den	MS	0.38
CPDM	AcCo	0.39
S1DZ	S1ULp	0.41
Den	CPDM	0.42
S1ULp	DI	0.43
CPDM	CPVM	0.43
M2	S1FL	0.44
CI	CPVL	0.44
AIV	CPVM	0.44
LSD	Tu	0.45
CI	AcCo	0.46
LSI	MS	0.46
cg2	S1ULp	0.47
CI	LSS	0.47
Den	CPVM	0.47

S1DZ	DI	0.5
LSI	Shi	0.5
GI	CPVM	0.51
LSS	AcSh	0.51
Pir	AcSh	0.52
M1	MS	0.52
cg1	M1	0.53
cg1	S1FL	0.53
CPDM	CPVL	0.53
Den	AcSh	0.53
CPDL	CPVL	0.55
LSI	LSV	0.56
LSV	Tu	0.56
S1DZ	GI	0.58
CPVM	AcCo	0.58
Den	CPVL	0.59
S1JO	VDB	0.59
Pir	VP	0.59
AID	AIV	0.6
CPDM	CPDL	0.6
cg2	M2	0.61
M1	AID	0.61
LSD	LSI	0.62
Pir	AcCo	0.63
CPDL	Shi	0.64
AcCo	AcSh	0.65
M2	S1j	0.68
AID	LSS	0.68
LSS	CPVM	0.68
Den	LSV	0.7
DI	LSV	0.72
M2	M1	0.73
cg1	S1j	0.74
CI	CPDL	0.77
AIV	AcSh	0.77
AcSh	VDB	0.77
LSD	Shi	0.78
CPDM	VDB	0.79
cg2	cg1	0.8
Shi	MS	0.81
CPVL	CPVM	0.84
CPVM	VDB	0.85
CI	VDB	0.86
LSS	LSV	0.88
AcCo	LSV	0.93
Ig	cg1	1
Ig	M2	1

Ig	M1	1
cg2	M1	1
Ig	S1FL	1
cg2	S1FL	1
Ig	S1j	1
cg2	S1j	1
Ig	S1JO	1
cg1	S1JO	1
M2	S1JO	1
M1	S1JO	1
S1FL	S1JO	1
S1j	S1JO	1
Ig	S1DZ	1
cg1	S1DZ	1
M2	S1DZ	1
S1FL	S1DZ	1
S1j	S1DZ	1
S1JO	S1DZ	1
Ig	S1ULp	1
cg1	S1ULp	1
M2	S1ULp	1
M1	S1ULp	1
S1FL	S1ULp	1
S1j	S1ULp	1
S1JO	S1ULp	1
Ig	GI	1
cg1	GI	1
M2	GI	1
S1FL	GI	1
S1j	GI	1
S1JO	GI	1
Ig	DI	1
cg1	DI	1
M2	DI	1
M1	DI	1
S1FL	DI	1
S1j	DI	1
S1JO	DI	1
Ig	AID	1
cg2	AID	1
cg1	AID	1
M2	AID	1
S1JO	AID	1
S1DZ	AID	1
S1ULp	AID	1
GI	AID	1
DI	AID	1

Ig	AIV	1
cg2	AIV	1
S1JO	AIV	1
S1DZ	AIV	1
S1ULp	AIV	1
GI	AIV	1
DI	AIV	1
Ig	CI	1
cg2	CI	1
cg1	CI	1
M2	CI	1
S1DZ	CI	1
S1ULp	CI	1
GI	CI	1
DI	CI	1
AID	CI	1
Ig	Pir	1
cg2	Pir	1
cg1	Pir	1
M2	Pir	1
M1	Pir	1
S1FL	Pir	1
S1j	Pir	1
S1JO	Pir	1
S1DZ	Pir	1
S1ULp	Pir	1
GI	Pir	1
DI	Pir	1
AIV	Pir	1
Ig	Den	1
cg2	Den	1
S1JO	Den	1
S1DZ	Den	1
S1ULp	Den	1
GI	Den	1
DI	Den	1
Pir	Den	1
Ig	LSS	1
cg2	LSS	1
S1JO	LSS	1
S1DZ	LSS	1
S1ULp	LSS	1
GI	LSS	1
DI	LSS	1
Ig	CPDM	1
cg1	CPDM	1
M2	CPDM	1

S1DZ	CPDM	1
S1ULp	CPDM	1
GI	CPDM	1
DI	CPDM	1
AID	CPDM	1
Ig	CPDL	1
cg2	CPDL	1
S1JO	CPDL	1
S1DZ	CPDL	1
S1ULp	CPDL	1
GI	CPDL	1
Pir	CPDL	1
Ig	CPVL	1
cg2	CPVL	1
S1DZ	CPVL	1
S1ULp	CPVL	1
DI	CPVL	1
Pir	CPVL	1
Ig	CPVM	1
cg2	CPVM	1
cg1	CPVM	1
M2	CPVM	1
S1DZ	CPVM	1
S1ULp	CPVM	1
DI	CPVM	1
AID	CPVM	1
CPDL	CPVM	1
Ig	AcCo	1
cg2	AcCo	1
cg1	AcCo	1
M2	AcCo	1
M1	AcCo	1
S1FL	AcCo	1
S1j	AcCo	1
S1JO	AcCo	1
S1DZ	AcCo	1
S1ULp	AcCo	1
GI	AcCo	1
DI	AcCo	1
AID	AcCo	1
AIV	AcCo	1
Den	AcCo	1
CPDL	AcCo	1
CPVL	AcCo	1
Ig	AcSh	1
cg2	AcSh	1
cg1	AcSh	1

M2	AcSh	1
S1JO	AcSh	1
S1DZ	AcSh	1
S1ULp	AcSh	1
GI	AcSh	1
DI	AcSh	1
AID	AcSh	1
Ig	LSD	1
cg2	LSD	1
cg1	LSD	1
M2	LSD	1
M1	LSD	1
S1FL	LSD	1
S1j	LSD	1
S1JO	LSD	1
S1DZ	LSD	1
S1ULp	LSD	1
GI	LSD	1
DI	LSD	1
AID	LSD	1
AIV	LSD	1
CI	LSD	1
Pir	LSD	1
Den	LSD	1
LSS	LSD	1
CPDM	LSD	1
CPDL	LSD	1
CPVL	LSD	1
CPVM	LSD	1
AcCo	LSD	1
AcSh	LSD	1
Ig	LSI	1
cg2	LSI	1
M1	LSI	1
S1FL	LSI	1
S1j	LSI	1
S1DZ	LSI	1
S1ULp	LSI	1
GI	LSI	1
DI	LSI	1
AID	LSI	1
AIV	LSI	1
CI	LSI	1
Pir	LSI	1
Den	LSI	1
LSS	LSI	1
CPDM	LSI	1

CPDL	LSI	1
CPVL	LSI	1
CPVM	LSI	1
AcCo	LSI	1
AcSh	LSI	1
Ig	LSV	1
cg2	LSV	1
cg1	LSV	1
M1	LSV	1
S1FL	LSV	1
S1j	LSV	1
S1JO	LSV	1
S1DZ	LSV	1
S1ULp	LSV	1
GI	LSV	1
AID	LSV	1
AIV	LSV	1
CI	LSV	1
Pir	LSV	1
CPDM	LSV	1
CPDL	LSV	1
CPVL	LSV	1
CPVM	LSV	1
AcSh	LSV	1
Ig	Shi	1
cg2	Shi	1
M1	Shi	1
S1FL	Shi	1
S1j	Shi	1
S1DZ	Shi	1
S1ULp	Shi	1
GI	Shi	1
AID	Shi	1
AIV	Shi	1
CI	Shi	1
Pir	Shi	1
Den	Shi	1
LSS	Shi	1
CPDM	Shi	1
CPVL	Shi	1
CPVM	Shi	1
AcCo	Shi	1
AcSh	Shi	1
LSV	Shi	1
Ig	MS	1
S1JO	MS	1
S1DZ	MS	1

S1ULp	MS	1
GI	MS	1
DI	MS	1
AID	MS	1
CI	MS	1
Pir	MS	1
LSS	MS	1
CPDM	MS	1
CPVM	MS	1
AcSh	MS	1
LSD	MS	1
LSV	MS	1
Ig	VDB	1
cg2	VDB	1
cg1	VDB	1
M2	VDB	1
M1	VDB	1
S1FL	VDB	1
S1j	VDB	1
S1DZ	VDB	1
S1ULp	VDB	1
GI	VDB	1
DI	VDB	1
AID	VDB	1
AIV	VDB	1
Pir	VDB	1
Den	VDB	1
LSS	VDB	1
CPDL	VDB	1
CPVL	VDB	1
LSD	VDB	1
LSI	VDB	1
LSV	VDB	1
Shi	VDB	1
MS	VDB	1
Ig	Icj	1
cg1	Icj	1
M2	Icj	1
M1	Icj	1
S1FL	Icj	1
S1j	Icj	1
AID	Icj	1
AIV	Icj	1
CI	Icj	1
Pir	Icj	1
Den	Icj	1
LSS	Icj	1

CPDM	Icj	1
CPVL	Icj	1
CPVM	Icj	1
AcCo	Icj	1
AcSh	Icj	1
LSD	Icj	1
LSI	Icj	1
LSV	Icj	1
Shi	Icj	1
MS	Icj	1
VDB	Icj	1
Ig	VP	1
cg2	VP	1
cg1	VP	1
M2	VP	1
M1	VP	1
S1FL	VP	1
S1j	VP	1
S1JO	VP	1
S1DZ	VP	1
GI	VP	1
DI	VP	1
AID	VP	1
AIV	VP	1
CI	VP	1
Den	VP	1
LSS	VP	1
CPDM	VP	1
CPDL	VP	1
CPVL	VP	1
CPVM	VP	1
AcSh	VP	1
LSD	VP	1

LSI	VP	1
LSV	VP	1
Shi	VP	1
Icj	VP	1
Ig	Tu	1
cg2	Tu	1
cg1	Tu	1
M2	Tu	1
M1	Tu	1
S1FL	Tu	1
S1j	Tu	1
S1JO	Tu	1
S1DZ	Tu	1
GI	Tu	1
DI	Tu	1
AID	Tu	1
AIV	Tu	1
CI	Tu	1
Pir	Tu	1
Den	Tu	1
LSS	Tu	1
CPDM	Tu	1
CPDL	Tu	1
CPVL	Tu	1
CPVM	Tu	1
AcCo	Tu	1
AcSh	Tu	1
LSI	Tu	1
Shi	Tu	1
VDB	Tu	1
Icj	Tu	1
VP	Tu	1

Supplementary Table S6. Results of permutation test to assess significant differences in pairs of edge weight between VEH/VEH and KET/VEH groups. The p-values for all possible pairs are reported, significant values were given in bold.

ROI	ROI	p-value
M1	AIV	**0.01**
S1FL	AIV	**0.01**
AIV	CPDL	**0.01**
Ig	LSI	**0.01**
S1j	AIV	**0.02**
S1FL	Den	**0.03**
S1j	Den	**0.03**
AIV	CPVL	**0.03**
Ig	DI	0.05
M1	LSS	0.05
DI	Icj	0.05
M1	Den	0.06
Den	CPVL	0.07
cg1	AIV	0.08
LSS	CPVL	0.08
AcCo	VDB	0.08
AIV	CPDM	0.09
DI	LSD	0.09
Ig	LSV	0.09
GI	Icj	0.09
M1	S1JO	0.1
Den	CPDL	0.1
Ig	LSD	0.1
M2	AIV	0.11
AIV	LSS	0.11
CPDM	CPDL	0.11
Ig	cg2	0.12
LSS	CPDL	0.12
CPDM	VDB	0.12
cg1	CPDL	0.13
S1FL	AID	0.14
M2	Icj	0.14
M1	AID	0.15
M2	Den	0.15
S1JO	LSI	0.15
cg1	Den	0.16
CPVM	AcCo	0.16
AIV	Tu	0.16
AID	CPDL	0.17
AIV	CPVM	0.17
S1ULp	Shi	0.17
S1j	AID	0.18
AIV	AcSh	0.18
S1j	Icj	0.18
S1ULp	GI	0.19
S1DZ	AID	0.19
AIV	Den	0.19
Ig	LSS	0.19
cg2	S1JO	0.2
S1j	CPDL	0.2
S1FL	Icj	0.2
S1ULp	DI	0.21
S1DZ	Icj	0.21
Ig	S1ULp	0.22
GI	CPVM	0.22
Ig	Shi	0.22
S1FL	LSS	0.23
AID	CPVL	0.23
CPDL	CPVM	0.23

AIV	MS	0.23
cg2	M1	0.24
cg1	S1ULp	0.25
CI	CPVM	0.25
S1DZ	GI	0.27
Den	LSS	0.27
S1j	CPVL	0.27
CPVL	CPVM	0.27
DI	LSV	0.27
S1JO	Shi	0.27
cg2	S1ULp	0.28
GI	DI	0.28
S1FL	CPVM	0.28
Den	CPVM	0.28
cg2	DI	0.29
AID	Pir	0.3
CPDM	AcSh	0.3
CPVM	AcSh	0.3
cg1	Icj	0.3
S1FL	CPDL	0.31
M1	CPVL	0.31
MS	Icj	0.31
AcSh	VP	0.31
S1j	Shi	0.33
M1	CI	0.34
AIV	CI	0.34
S1j	LSS	0.34
CI	AcSh	0.34
S1ULp	LSV	0.34
M1	S1j	0.35
S1DZ	Pir	0.36
cg1	CPVL	0.36

CPDM	CPVM	0.36
Den	MS	0.36
Ig	cg1	0.37
Ig	CPDL	0.37
S1DZ	S1ULp	0.38
Den	CPDM	0.39
M2	S1FL	0.4
cg2	GI	0.4
VDB	VP	0.4
CPDL	MS	0.41
LSS	CPVM	0.42
S1ULp	AcSh	0.42
S1ULp	LSI	0.42
CPDL	LSI	0.42
S1FL	Shi	0.42
M2	CPDL	0.43
cg1	VP	0.44
S1ULp	VP	0.45
DI	LSI	0.46
M2	S1ULp	0.47
S1FL	S1ULp	0.47
S1FL	CI	0.47
M2	LSS	0.47
CPDL	CPVL	0.47
CPVL	MS	0.47
AID	Den	0.49
AcSh	Shi	0.49
GI	VDB	0.49
CI	AcCo	0.5
cg1	M2	0.51
M1	CPVM	0.51
M2	LSI	0.51

Pir	VP	0.51
cg1	LSS	0.52
M1	CPDM	0.52
S1j	CPVM	0.52
CPDM	AcCo	0.52
S1j	MS	0.52
Shi	Icj	0.52
S1j	AcSh	0.53
M1	S1FL	0.54
LSS	CPDM	0.54
M1	CPDL	0.54
S1FL	CPVL	0.54
Pir	AcSh	0.55
LSS	LSV	0.55
CI	Pir	0.56
LSS	AcSh	0.56
AID	LSD	0.57
cg1	MS	0.57
S1j	CPDM	0.59
S1DZ	DI	0.6
CPDL	LSV	0.6
LSD	LSV	0.6
AcSh	VDB	0.6
M1	MS	0.61
S1j	S1ULp	0.62
LSS	LSI	0.62
LSV	Tu	0.62
M2	M1	0.63
DI	AID	0.63
CI	CPVL	0.63
cg2	MS	0.63
AID	LSS	0.64

M1	AcSh	0.64
S1ULp	MS	0.64
M2	S1j	0.65
S1j	CI	0.65
M2	CPVL	0.66
S1FL	CPDM	0.67
cg1	LSV	0.67
S1FL	MS	0.67
cg2	cg1	0.68
cg1	M1	0.68
cg2	S1DZ	0.68
CI	CPDM	0.68
cg2	M2	0.7
cg1	AcSh	0.7
CI	VDB	0.7
cg2	Icj	0.7
AID	AIV	0.71
Pir	CPVM	0.71
LSI	LSV	0.71
LSD	Shi	0.71
S1FL	AcSh	0.72
cg1	Shi	0.72
S1ULp	Icj	0.73
Den	AcSh	0.74
Pir	CPDM	0.75
LSS	MS	0.75
cg2	Pir	0.81
LSD	Tu	0.81
Shi	VP	0.82
CPDL	AcSh	0.83
CPVL	AcSh	0.84
LSI	Shi	0.84

S1DZ	LSD	0.88
LSI	MS	0.89
Pir	CPVL	0.9
Ig	M2	1
Ig	M1	1
Ig	S1FL	1
cg2	S1FL	1
cg1	S1FL	1
Ig	S1j	1
cg2	S1j	1
cg1	S1j	1
S1FL	S1j	1
Ig	S1JO	1
cg1	S1JO	1
M2	S1JO	1
S1FL	S1JO	1
S1j	S1JO	1
Ig	S1DZ	1
cg1	S1DZ	1
M2	S1DZ	1
M1	S1DZ	1
S1FL	S1DZ	1
S1j	S1DZ	1
S1JO	S1DZ	1
M1	S1ULp	1
S1JO	S1ULp	1
Ig	GI	1
cg1	GI	1
M2	GI	1
M1	GI	1
S1FL	GI	1
S1j	GI	1

S1JO	GI	1
cg1	DI	1
M2	DI	1
M1	DI	1
S1FL	DI	1
S1j	DI	1
S1JO	DI	1
Ig	AID	1
cg2	AID	1
cg1	AID	1
M2	AID	1
S1JO	AID	1
S1ULp	AID	1
GI	AID	1
Ig	AIV	1
cg2	AIV	1
S1JO	AIV	1
S1DZ	AIV	1
S1ULp	AIV	1
GI	AIV	1
DI	AIV	1
Ig	CI	1
cg2	CI	1
cg1	CI	1
M2	CI	1
S1JO	CI	1
S1DZ	CI	1
S1ULp	CI	1
GI	CI	1
DI	CI	1
AID	CI	1
Ig	Pir	1

cg1	Pir	1
M2	Pir	1
M1	Pir	1
S1FL	Pir	1
S1j	Pir	1
S1JO	Pir	1
S1ULp	Pir	1
GI	Pir	1
DI	Pir	1
AIV	Pir	1
Ig	Den	1
cg2	Den	1
S1JO	Den	1
S1DZ	Den	1
S1ULp	Den	1
GI	Den	1
DI	Den	1
CI	Den	1
Pir	Den	1
cg2	LSS	1
S1JO	LSS	1
S1DZ	LSS	1
S1ULp	LSS	1
GI	LSS	1
DI	LSS	1
CI	LSS	1
Pir	LSS	1
Ig	CPDM	1
cg2	CPDM	1
cg1	CPDM	1
M2	CPDM	1
S1JO	CPDM	1

S1DZ	CPDM	1
S1ULp	CPDM	1
GI	CPDM	1
DI	CPDM	1
AID	CPDM	1
cg2	CPDL	1
S1JO	CPDL	1
S1DZ	CPDL	1
S1ULp	CPDL	1
GI	CPDL	1
DI	CPDL	1
CI	CPDL	1
Pir	CPDL	1
Ig	CPVL	1
cg2	CPVL	1
S1JO	CPVL	1
S1DZ	CPVL	1
S1ULp	CPVL	1
GI	CPVL	1
DI	CPVL	1
CPDM	CPVL	1
Ig	CPVM	1
cg2	CPVM	1
cg1	CPVM	1
M2	CPVM	1
S1JO	CPVM	1
S1DZ	CPVM	1
S1ULp	CPVM	1
DI	CPVM	1
AID	CPVM	1
Ig	AcCo	1
cg2	AcCo	1

cg1	AcCo	1
M2	AcCo	1
M1	AcCo	1
S1FL	AcCo	1
S1j	AcCo	1
S1JO	AcCo	1
S1DZ	AcCo	1
S1ULp	AcCo	1
GI	AcCo	1
DI	AcCo	1
AID	AcCo	1
AIV	AcCo	1
Pir	AcCo	1
Den	AcCo	1
LSS	AcCo	1
CPDL	AcCo	1
CPVL	AcCo	1
Ig	AcSh	1
cg2	AcSh	1
M2	AcSh	1
S1JO	AcSh	1
S1DZ	AcSh	1
GI	AcSh	1
DI	AcSh	1
AID	AcSh	1
AcCo	AcSh	1
cg2	LSD	1
cg1	LSD	1
M2	LSD	1
M1	LSD	1
S1FL	LSD	1
S1j	LSD	1

S1JO	LSD	1
S1ULp	LSD	1
GI	LSD	1
AIV	LSD	1
CI	LSD	1
Pir	LSD	1
Den	LSD	1
LSS	LSD	1
CPDM	LSD	1
CPDL	LSD	1
CPVL	LSD	1
CPVM	LSD	1
AcCo	LSD	1
AcSh	LSD	1
cg2	LSI	1
cg1	LSI	1
M1	LSI	1
S1FL	LSI	1
S1j	LSI	1
S1DZ	LSI	1
GI	LSI	1
AID	LSI	1
AIV	LSI	1
CI	LSI	1
Pir	LSI	1
Den	LSI	1
CPDM	LSI	1
CPVL	LSI	1
CPVM	LSI	1
AcCo	LSI	1
AcSh	LSI	1
LSD	LSI	1

cg2	LSV	1
M2	LSV	1
M1	LSV	1
S1FL	LSV	1
S1j	LSV	1
S1JO	LSV	1
S1DZ	LSV	1
GI	LSV	1
AID	LSV	1
AIV	LSV	1
CI	LSV	1
Pir	LSV	1
Den	LSV	1
CPDM	LSV	1
CPVL	LSV	1
CPVM	LSV	1
AcCo	LSV	1
AcSh	LSV	1
cg2	Shi	1
M2	Shi	1
M1	Shi	1
S1DZ	Shi	1
GI	Shi	1
DI	Shi	1
AID	Shi	1
AIV	Shi	1
CI	Shi	1
Pir	Shi	1
Den	Shi	1
LSS	Shi	1
CPDM	Shi	1
CPDL	Shi	1

CPVL	Shi	1
CPVM	Shi	1
AcCo	Shi	1
LSV	Shi	1
Ig	MS	1
M2	MS	1
S1JO	MS	1
S1DZ	MS	1
GI	MS	1
DI	MS	1
AID	MS	1
CI	MS	1
Pir	MS	1
CPDM	MS	1
CPVM	MS	1
AcCo	MS	1
AcSh	MS	1
LSD	MS	1
LSV	MS	1
Shi	MS	1
Ig	VDB	1
cg2	VDB	1
cg1	VDB	1
M2	VDB	1
M1	VDB	1
S1FL	VDB	1
S1j	VDB	1
S1JO	VDB	1
S1DZ	VDB	1
S1ULp	VDB	1
DI	VDB	1
AID	VDB	1

AIV	VDB	1
Pir	VDB	1
Den	VDB	1
LSS	VDB	1
CPDL	VDB	1
CPVL	VDB	1
CPVM	VDB	1
LSD	VDB	1
LSI	VDB	1
LSV	VDB	1
Shi	VDB	1
MS	VDB	1
Ig	Icj	1
M1	Icj	1
S1JO	Icj	1
AID	Icj	1
AIV	Icj	1
CI	Icj	1
Pir	Icj	1
Den	Icj	1
LSS	Icj	1
CPDM	Icj	1
CPDL	Icj	1
CPVL	Icj	1
CPVM	Icj	1
AcCo	Icj	1
AcSh	Icj	1
LSD	Icj	1
LSI	Icj	1
LSV	Icj	1
VDB	Icj	1
Ig	VP	1

cg2	VP	1
M2	VP	1
M1	VP	1
S1FL	VP	1
S1j	VP	1
S1JO	VP	1
S1DZ	VP	1
GI	VP	1
DI	VP	1
AID	VP	1
AIV	VP	1
CI	VP	1
Den	VP	1
LSS	VP	1
CPDM	VP	1
CPDL	VP	1
CPVL	VP	1
CPVM	VP	1
AcCo	VP	1
LSD	VP	1
LSI	VP	1
LSV	VP	1
MS	VP	1
Icj	VP	1
Ig	Tu	1
cg2	Tu	1
cg1	Tu	1
M2	Tu	1
M1	Tu	1
S1FL	Tu	1
S1j	Tu	1
S1JO	Tu	1

S1DZ	Tu	1
S1ULp	Tu	1
GI	Tu	1
DI	Tu	1
AID	Tu	1
CI	Tu	1
Pir	Tu	1
Den	Tu	1
LSS	Tu	1
CPDM	Tu	1
CPDL	Tu	1

CPVL	Tu	1
CPVM	Tu	1
AcCo	Tu	1
AcSh	Tu	1
LSI	Tu	1
Shi	Tu	1
MS	Tu	1
VDB	Tu	1
Icj	Tu	1
VP	Tu	1

Supplementary Table S7. Results of permutation test to assess significant differences in pairs of edge weight between KET/VEH and KET/ASE groups. The p-values for all possible pairs are reported, significant values were given in bold.

ROI	ROI	p-value
Ig	LSI	**0.01**
Ig	CPDL	**0.04**
Ig	LSD	**0.04**
Ig	LSV	**0.04**
Ig	S1ULp	**<0.05**
Ig	CI	**<0.05**
Ig	CPVL	**<0.05**
Ig	LSS	0.06
Ig	CPVM	0.06
CI	Tu	0.06
Ig	cg1	0.08
S1JO	CI	0.1
LSS	CPDL	0.1
S1JO	CPVM	0.1
Ig	DI	0.12
CPDM	CPDL	0.12
CPVM	AcCo	0.13
S1j	Den	0.14
AcSh	VP	0.14
CPVL	Tu	0.14
M2	AIV	0.15
CPVL	CPVM	0.15
LSS	LSD	0.15
S1JO	CPVL	0.16
M1	S1JO	0.17
LSD	VDB	0.17
AcSh	Icj	0.17
M1	AIV	0.18
S1j	Pir	0.18
AcSh	VDB	0.18
AcCo	Icj	0.19
CPDL	CPVM	0.21
AcCo	Shi	0.21
LSD	Icj	0.21
CPVM	Tu	0.21
AcCo	VDB	0.22
Shi	VP	0.22
CPDM	CPVL	0.23
CPVL	LSV	0.23
LSD	LSV	0.23
S1ULp	Shi	0.23
S1FL	AIV	0.24
S1JO	AcCo	0.24
S1JO	AcSh	0.24
CPVL	LSD	0.24
Ig	Shi	0.24
CPDL	LSV	0.26
M1	S1FL	0.27
cg1	S1ULp	0.27
S1ULp	GI	0.27
Ig	Tu	0.27
S1DZ	CPDM	0.28
cg1	Shi	0.28
LSD	Shi	0.28
S1JO	CPDL	0.29
DI	LSD	0.29
S1ULp	DI	0.3
S1JO	LSV	0.3
AcCo	VP	0.31
S1j	Icj	0.32
CPVM	AcSh	0.33
MS	Icj	0.33
S1JO	Tu	0.33
LSS	AcSh	0.34
DI	LSV	0.34
LSS	LSV	0.34
LSI	Icj	0.34
S1FL	Icj	0.35
LSI	LSV	0.36

CI	CPVL	0.37
M2	Icj	0.37
VDB	Icj	0.37
Ig	Icj	0.38
AcCo	Tu	0.38
Ig	S1JO	0.39
CI	CPDM	0.39
GI	CPVM	0.39
GI	AID	0.4
CI	Den	0.4
Den	CPDM	0.4
S1FL	MS	0.4
CPVM	Icj	0.4
AcSh	Tu	0.4
Ig	AcSh	0.41
AcCo	LSD	0.41
M2	MS	0.41
Ig	AcCo	0.42
S1DZ	LSD	0.42
CPVM	LSV	0.42
CPDL	Tu	0.42
M1	AID	0.44
CI	LSS	0.44
AID	CPDM	0.44
LSI	Shi	0.44
LSS	VDB	0.44
LSS	AcCo	0.45
CI	VDB	0.45
LSV	Tu	0.45
AIV	Den	0.46
AcCo	AcSh	0.47
GI	DI	0.48
S1j	CPDM	0.48
AcSh	LSD	0.48
S1j	Shi	0.48
S1JO	LSD	0.49
S1ULp	AID	0.5

M1	Den	0.5
Pir	Den	0.5
S1JO	Icj	0.5
GI	AIV	0.51
M2	Den	0.51
M1	Pir	0.52
CI	LSV	0.52
LSD	Tu	0.52
S1j	AIV	0.53
LSV	Shi	0.53
S1ULp	MS	0.53
S1JO	VDB	0.54
LSS	Icj	0.54
DI	AID	0.55
S1ULp	CPDM	0.55
Pir	CPDM	0.55
CPVL	AcCo	0.55
S1ULp	LSV	0.55
M2	M1	0.56
S1DZ	GI	0.56
Pir	CPVL	0.56
CPVL	VDB	0.56
cg2	cg1	0.57
M2	S1j	0.57
CPVL	AcSh	0.57
AID	LSD	0.57
S1ULp	Icj	0.57
S1j	S1ULp	0.58
M2	Pir	0.58
AID	Pir	0.58
CI	LSD	0.58
cg1	MS	0.58
cg1	M2	0.59
cg2	M1	0.59
cg2	S1DZ	0.59
S1FL	DI	0.59
M2	AID	0.59

S1DZ	Den	0.59
S1j	MS	0.59
cg1	S1FL	0.6
S1FL	AID	0.6
Shi	MS	0.6
VDB	Tu	0.6
S1DZ	CPDL	0.61
Ig	VDB	0.63
Icj	Tu	0.63
cg1	VP	0.64
AIV	Tu	0.64
M2	DI	0.65
M1	DI	0.65
S1FL	Pir	0.65
AcCo	LSI	0.66
CPVL	Icj	0.66
cg2	S1JO	0.67
DI	CPDM	0.67
AcSh	LSI	0.67
S1ULp	AIV	0.68
cg2	CPDM	0.68
CPVM	LSD	0.69
S1DZ	AID	0.7
cg1	LSI	0.7
S1ULp	LSI	0.7
M2	Shi	0.7
M1	S1j	0.71
S1FL	Den	0.71
S1ULp	VP	0.71
AID	AIV	0.72
cg1	AcSh	0.73
S1ULp	AcSh	0.73
M1	GI	0.74
DI	AIV	0.74
CI	AcSh	0.74
GI	CPDM	0.75
cg2	MS	0.75

CI	AcCo	0.76
cg1	LSV	0.76
DI	LSI	0.77
GI	VDB	0.77
LSI	VDB	0.77
cg2	Pir	0.78
cg1	Icj	0.78
cg1	S1j	0.79
M2	GI	0.79
S1FL	GI	0.79
AIV	Pir	0.8
S1j	S1DZ	0.81
S1DZ	S1ULp	0.81
S1FL	Shi	0.82
CI	CPVM	0.83
S1FL	S1j	0.84
LSS	LSI	0.86
M1	S1ULp	0.87
Shi	VDB	0.87
CI	Icj	0.87
Ig	cg2	1
Ig	M2	1
cg2	M2	1
Ig	M1	1
cg1	M1	1
Ig	S1FL	1
cg2	S1FL	1
M2	S1FL	1
Ig	S1j	1
cg2	S1j	1
cg1	S1JO	1
M2	S1JO	1
S1FL	S1JO	1
S1j	S1JO	1
Ig	S1DZ	1
cg1	S1DZ	1
M2	S1DZ	1

M1	S1DZ	1
S1FL	S1DZ	1
S1JO	S1DZ	1
cg2	S1ULp	1
M2	S1ULp	1
S1FL	S1ULp	1
S1JO	S1ULp	1
Ig	GI	1
cg2	GI	1
cg1	GI	1
S1j	GI	1
S1JO	GI	1
cg2	DI	1
cg1	DI	1
S1j	DI	1
S1JO	DI	1
S1DZ	DI	1
Ig	AID	1
cg2	AID	1
cg1	AID	1
S1j	AID	1
S1JO	AID	1
Ig	AIV	1
cg2	AIV	1
cg1	AIV	1
S1JO	AIV	1
S1DZ	AIV	1
cg2	CI	1
cg1	CI	1
M2	CI	1
M1	CI	1
S1FL	CI	1
S1j	CI	1
S1DZ	CI	1
S1ULp	CI	1
GI	CI	1
DI	CI	1

AID	CI	1
AIV	CI	1
Ig	Pir	1
cg1	Pir	1
S1JO	Pir	1
S1DZ	Pir	1
S1ULp	Pir	1
GI	Pir	1
DI	Pir	1
CI	Pir	1
Ig	Den	1
cg2	Den	1
cg1	Den	1
S1JO	Den	1
S1ULp	Den	1
GI	Den	1
DI	Den	1
AID	Den	1
cg2	LSS	1
cg1	LSS	1
M2	LSS	1
M1	LSS	1
S1FL	LSS	1
S1j	LSS	1
S1JO	LSS	1
S1DZ	LSS	1
S1ULp	LSS	1
GI	LSS	1
DI	LSS	1
AID	LSS	1
AIV	LSS	1
Pir	LSS	1
Den	LSS	1
Ig	CPDM	1
cg1	CPDM	1
M2	CPDM	1
M1	CPDM	1

S1FL	CPDM	1
S1JO	CPDM	1
AIV	CPDM	1
LSS	CPDM	1
cg2	CPDL	1
cg1	CPDL	1
M2	CPDL	1
M1	CPDL	1
S1FL	CPDL	1
S1j	CPDL	1
S1ULp	CPDL	1
GI	CPDL	1
DI	CPDL	1
AID	CPDL	1
AIV	CPDL	1
CI	CPDL	1
Pir	CPDL	1
Den	CPDL	1
cg2	CPVL	1
cg1	CPVL	1
M2	CPVL	1
M1	CPVL	1
S1FL	CPVL	1
S1j	CPVL	1
S1DZ	CPVL	1
S1ULp	CPVL	1
GI	CPVL	1
DI	CPVL	1
AID	CPVL	1
AIV	CPVL	1
Den	CPVL	1
LSS	CPVL	1
CPDL	CPVL	1
cg2	CPVM	1
cg1	CPVM	1
M2	CPVM	1
M1	CPVM	1

S1FL	CPVM	1
S1j	CPVM	1
S1DZ	CPVM	1
S1ULp	CPVM	1
DI	CPVM	1
AID	CPVM	1
AIV	CPVM	1
Pir	CPVM	1
Den	CPVM	1
LSS	CPVM	1
CPDM	CPVM	1
cg2	AcCo	1
cg1	AcCo	1
M2	AcCo	1
M1	AcCo	1
S1FL	AcCo	1
S1j	AcCo	1
S1DZ	AcCo	1
S1ULp	AcCo	1
GI	AcCo	1
DI	AcCo	1
AID	AcCo	1
AIV	AcCo	1
Pir	AcCo	1
Den	AcCo	1
CPDM	AcCo	1
CPDL	AcCo	1
cg2	AcSh	1
M2	AcSh	1
M1	AcSh	1
S1FL	AcSh	1
S1j	AcSh	1
S1DZ	AcSh	1
GI	AcSh	1
DI	AcSh	1
AID	AcSh	1
AIV	AcSh	1

Pir	AcSh	1
Den	AcSh	1
CPDM	AcSh	1
CPDL	AcSh	1
cg2	LSD	1
cg1	LSD	1
M2	LSD	1
M1	LSD	1
S1FL	LSD	1
S1j	LSD	1
S1ULp	LSD	1
GI	LSD	1
AIV	LSD	1
Pir	LSD	1
Den	LSD	1
CPDM	LSD	1
CPDL	LSD	1
cg2	LSI	1
M2	LSI	1
M1	LSI	1
S1FL	LSI	1
S1j	LSI	1
S1JO	LSI	1
S1DZ	LSI	1
GI	LSI	1
AID	LSI	1
AIV	LSI	1
CI	LSI	1
Pir	LSI	1
Den	LSI	1
CPDM	LSI	1
CPDL	LSI	1
CPVL	LSI	1
CPVM	LSI	1
LSD	LSI	1
cg2	LSV	1
M2	LSV	1

M1	LSV	1
S1FL	LSV	1
S1j	LSV	1
S1DZ	LSV	1
GI	LSV	1
AID	LSV	1
AIV	LSV	1
Pir	LSV	1
Den	LSV	1
CPDM	LSV	1
AcCo	LSV	1
AcSh	LSV	1
cg2	Shi	1
M1	Shi	1
S1JO	Shi	1
S1DZ	Shi	1
GI	Shi	1
DI	Shi	1
AID	Shi	1
AIV	Shi	1
CI	Shi	1
Pir	Shi	1
Den	Shi	1
LSS	Shi	1
CPDM	Shi	1
CPDL	Shi	1
CPVL	Shi	1
CPVM	Shi	1
AcSh	Shi	1
Ig	MS	1
M1	MS	1
S1JO	MS	1
S1DZ	MS	1
GI	MS	1
DI	MS	1
AID	MS	1
AIV	MS	1

CI	MS	1
Pir	MS	1
Den	MS	1
LSS	MS	1
CPDM	MS	1
CPDL	MS	1
CPVL	MS	1
CPVM	MS	1
AcCo	MS	1
AcSh	MS	1
LSD	MS	1
LSI	MS	1
LSV	MS	1
cg2	VDB	1
cg1	VDB	1
M2	VDB	1
M1	VDB	1
S1FL	VDB	1
S1j	VDB	1
S1DZ	VDB	1
S1ULp	VDB	1
DI	VDB	1
AID	VDB	1
AIV	VDB	1
Pir	VDB	1
Den	VDB	1
CPDM	VDB	1
CPDL	VDB	1
CPVM	VDB	1
LSV	VDB	1
MS	VDB	1
cg2	Icj	1
M1	Icj	1
S1DZ	Icj	1
GI	Icj	1
DI	Icj	1
AID	Icj	1

AIV	Icj	1
Pir	Icj	1
Den	Icj	1
CPDM	Icj	1
CPDL	Icj	1
LSV	Icj	1
Shi	Icj	1
Ig	VP	1
cg2	VP	1
M2	VP	1
M1	VP	1
S1FL	VP	1
S1j	VP	1
S1JO	VP	1
S1DZ	VP	1
GI	VP	1
DI	VP	1
AID	VP	1
AIV	VP	1
CI	VP	1
Pir	VP	1
Den	VP	1
LSS	VP	1
CPDM	VP	1
CPDL	VP	1
CPVL	VP	1
CPVM	VP	1
LSD	VP	1
LSI	VP	1
LSV	VP	1
MS	VP	1
VDB	VP	1
Icj	VP	1
cg2	Tu	1
cg1	Tu	1
M2	Tu	1
M1	Tu	1

S1FL	Tu	1
S1j	Tu	1
S1DZ	Tu	1
S1ULp	Tu	1
GI	Tu	1
DI	Tu	1
AID	Tu	1
Pir	Tu	1

Den	Tu	1
LSS	Tu	1
CPDM	Tu	1
LSI	Tu	1
Shi	Tu	1
MS	Tu	1
VP	Tu	1

Supplementary Table S8. Nodes degree and betweenness centrality are reported for each group of treatment.

	**VEH/VEH**		**VEH/ASE**		**KET/VEH**		**KET/ASE**	
	Degree	Betweenness	Degree	Betweenness	Degree	Betweenness	Degree	Betweenness
AcCo	11	38	4	65	1	0	9	11
AcSh	11	9	2	17	6	52	6	5
AID	7	17	4	45	4	43	14	74
AIV	12	19	1	0	0	/	12	39
cg1	10	5	0	/	6	23	1	0
cg2	8	5	2	0	0	/	8	3
CI	12	4	1	0	3	14	9	16
CPDL	13	18	2	0	0	/	7	23
CPDM	12	16	3	96	2	3	15	40
CPVL	13	74	3	51	2	9	10	15
CPVM	13	27	3	112	2	0	11	30
Den	14	34	3	88	2	0	5	6
DI	8	35	2	20	5	37	11	18
GI	5	6	4	35	5	42	11	14
Icj	7	14	0	/	7	18	8	7
Ig	2	0	0	/	6	22	4	2
LSD	3	0	2	25	4	27	8	17
LSI	5	4	2	18	4	16	2	0
LSS	11	24	3	61	2	0	4	1
LSV	4	7	3	85	4	47	9	14
M1	8	7	3	44	5	31	14	20
M2	12	5	2	24	7	14	12	18
MS	4	12	2	10	6	38	0	/
Pir	1	0	2	17	3	17	3	30
S1DZ	9	24	2	34	3	0	11	14
S1FL	13	15	1	0	7	51	6	9
S1j	13	69	1	0	3	16	13	11
S1JO	0	/	3	50	1	0	11	7
S1ULp	4	0	0	/	8	60	11	13

Shi	5	11	4	16	6	46	6	6
Tu	2	5	3	85	3	29	8	41
VDB	4	5	1	0	0	/	6	9
VP	2	4	0	/	3	19	5	0

Supplementary Table S9. The p-values computed after permutation testing are reported to highlight differences in betweenness centrality among groups.

	VEH/VEH *vs* VEH/ASE	VEH/VEH *vs* KET/VEH	KET/VEH *vs* KET/ASE
Ig	1	0.074	0.032
cg2	0.017	0.118	0.712
cg1	0.088	0.154	0.045
M2	0.088	0.022	0.809
M1	0.812	0.188	0.823
S1FL	0.64	0.148	0.915
S1j	0.631	0.37	0.457
S1JO	0.086	1	0.255
S1DZ	1	0.474	0.009
S1ULp	0.938	0.138	0.099
GI	0.617	1	0.479
DI	0.196	0.188	0.173
AID	1	0.241	0.414
AIV	0.692	0.245	0.838
CI	0.293	0.457	0.168
Pir	0.668	0.099	0.04
Den	0.582	0.262	0.378
LSS	0.344	0.86	0.427
CPDM	0.407	0.39	0.009
CPDL	0.089	0.159	0.056
CPVL	0.311	0.29	0.325
CPVM	0.459	0.196	0.414
AcCo	0.347	0.561	0.455
AcSh	0.497	0.629	0.545
LSD	0.386	0.354	0.102
LSI	0.192	0.784	0.067
LSV	0.031	0.271	0.017
Shi	0.66	0.733	0.035
MS	0.455	0.042	1
VDB	0.585	0.463	0.525

Icj	0.338	1	0.707
VP	0.596	0.473	0.367
Tu	0.62	1	0.5

Supplementary Table S10. The p-values computed after permutation testing are reported to highlight differences in nodes degree between groups.

	VEH/VEH *vs* VEH/ASE	VEH/VEH *vs* KET/VEH	KET/VEH *vs* KET/ASE
Ig	0.867	0.355	0.736
cg2	0.183	0.57	0.964
cg1	0.056	0.958	0.598
M2	0.101	0.437	0.749
M1	0.134	0.22	0.352
S1FL	0.02	0.34	0.713
S1j	0.094	0.46	0.979
S1JO	0.234	1	0.392
S1DZ	0.61	0.957	0.684
S1ULp	0.621	0.504	0.869
GI	0.962	0.708	0.744
DI	0.83	0.922	0.74
AID	0.35	0.687	0.531
AIV	0.05	0.082	0.463
CI	0.013	0.477	0.826
Pir	0.829	0.954	0.763
Den	0.02	0.164	0.621
LSS	0.057	0.445	0.758
CPDM	0.04	0.224	0.385
CPDL	0.018	0.21	0.393
CPVL	0.024	0.091	0.128
CPVM	0.16	0.162	0.181
AcCo	0.707	0.531	0.071
AcSh	0.053	0.628	0.3
LSD	0.425	0.873	0.303
LSI	0.245	0.922	0.893
LSV	0.868	0.724	0.592
Shi	0.369	0.487	0.538
MS	0.107	0.598	0.622
VDB	0.564	0.695	0.376

Icj	0.486	0.89	0.379
VP	0.889	0.852	0.687
Tu	1	0.937	0.405
